# Structure elucidation of the redox cofactor mycofactocin reveals oligo-glycosylation by MftF†
†Electronic supplementary information (ESI) available: ESI Appendix: materials and methods, supplementary figures, supplementary results and discussion (.PDF). Data Set 1: ^13^C-labeled compounds (.xlsx). Data Set 2: comparative metabolomics comparing *M. smegmatis* WT, mutant and complement strains (.xlsx). Data Set 3: comparative metabolomics of *M. smegmatis* WT treated with glucose or ethanol (.xlsx). Data Set 4: activity-based metabolic profiling (.xlsx). See DOI: 10.1039/d0sc01172j


**DOI:** 10.1039/d0sc01172j

**Published:** 2020-04-23

**Authors:** Luis Peña-Ortiz, Ana Patrícia Graça, Huijuan Guo, Daniel Braga, Tobias G. Köllner, Lars Regestein, Christine Beemelmanns, Gerald Lackner

**Affiliations:** a Junior Research Group Synthetic Microbiology , Leibniz Institute for Natural Product Research and Infection Biology (HKI) , Beutenbergstr. 11a , 07745 Jena , Germany . Email: gerald.lackner@leibniz-hki.de; b Friedrich Schiller University , Beutenbergstr. 11a , 07745 Jena , Germany; c Junior Research Group Chemical Biology of Microbe-Host Interactions , Leibniz Institute for Natural Product Research and Infection Biology (HKI) , Beutenbergstr. 11a , 07745 Jena , Germany; d Department of Biochemistry , Max Planck Institute for Chemical Ecology , Hans-Knöll-Str. 8 , 07745 Jena , Germany; e Bio Pilot Plant , Leibniz Institute for Natural Product Research and Infection Biology (HKI) , Beutenbergstr. 11a , 07745 Jena , Germany

## Abstract

Mycofactocin (MFT) is a redox cofactor belonging to the family of ribosomally synthesized and post-translationally modified peptides (RiPPs) and is involved in alcohol metabolism of mycobacteria including *Mycobacterium tuberculosis*. A preliminary biosynthetic model had been established by bioinformatics and *in vitro* studies, while the structure of natural MFT and key biosynthetic steps remained elusive. Here, we report the discovery of glycosylated MFT by ^13^C-labeling metabolomics and establish a model of its biosynthesis in *Mycolicibacterium smegmatis*. Extensive structure elucidation including NMR revealed that MFT is decorated with up to nine β-1,4-linked glucose residues including 2-*O*-methylglucose. Dissection of biosynthetic genes demonstrated that the oligoglycosylation is catalyzed by the glycosyltransferase MftF. Furthermore, we confirm the redox cofactor function of glycosylated MFTs by activity-based metabolic profiling using the carveol dehydrogenase LimC and show that the MFT pool expands during cultivation on ethanol. Our results will guide future studies into the biochemical functions and physiological roles of MFT in bacteria.

## Abstract 2

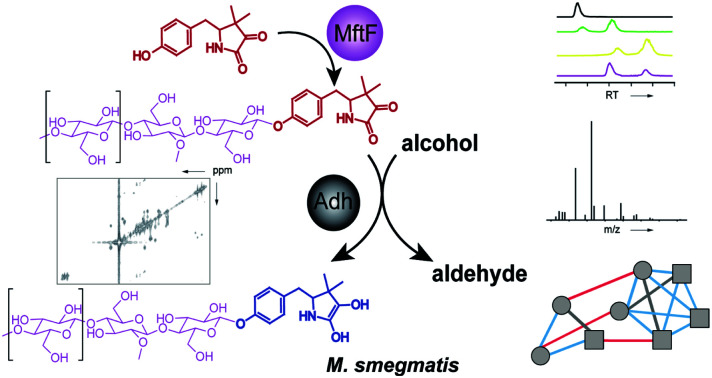
Metabolomics-driven discovery of the novel cofactor mycofactocin in mycobacteria revealed glycosylation with a cellulose-like sugar chain, regulation in response to ethanol and redox-activity.

## Introduction

Coenzymes are small molecules indispensable for the catalytic activity of many enzymes. While coenzymes like NAD^+^ or FAD are ubiquitous in nature and are essential for the core metabolism of all forms of life, specialized cofactors like pyrroloquinoline quinone (PQQ)^1^ and coenzyme F_420_ 2 are restricted to certain microbial phyla, but typically involved in extraordinary metabolic processes like methylotrophy, methanogenesis, or detoxification processes. Moreover, specialized cofactors serve as model systems for the evolution of cofactors and their co-evolution with their associated enzyme families. They can be regarded as examples of low-molecular weight natural products that modify, extend or enhance microbial metabolism. Mycobacteria are particularly rich in unusual redox cofactors and antioxidants that contribute to redox balance and metabolic plasticity. For instance, mycothiol^3^,^4^ or ergothioneine^5^ protect *Mycobacterium tuberculosis* from oxidative stress and support detoxification pathways. Coenzyme F_420_ is involved, *e.g.*, in cell wall biosynthesis^6^ or defense against nitrosative stress in mycobacteria.^7^ Moreover, some antimycobacterial drugs like pretomanid^8^ are administered as prodrugs and will only develop bioactivity after biotransformation by a coenzyme F_420_-dependent reductase.^9^ Mycofactocin (MFT) is a putative redox-cofactor whose existence has been postulated on the basis of comparative genomics and bioinformatics.^10^,^11^ Its molecular identity and structure, however, have remained elusive to date. The MFT biosynthetic gene cluster is highly conserved and widespread among mycobacteria. The inactivation of the MFT gene locus in the model species *Mycolicibacterium smegmatis* (synonym: *Mycobacterium smegmatis*) as well as *M. tuberculosis* resulted in the inability of the mutants to utilize ethanol as a sole source of carbon and further disturbances of mycobacterial redox homeostasis were revealed.^12^ Involvement of MFT in methanol metabolism was reported as well.^13^ These recent results strongly support the hypothesis that MFT is a redox cofactor and might represent a fitness factor of mycobacteria during some stages of infection.

**Fig. 1:**
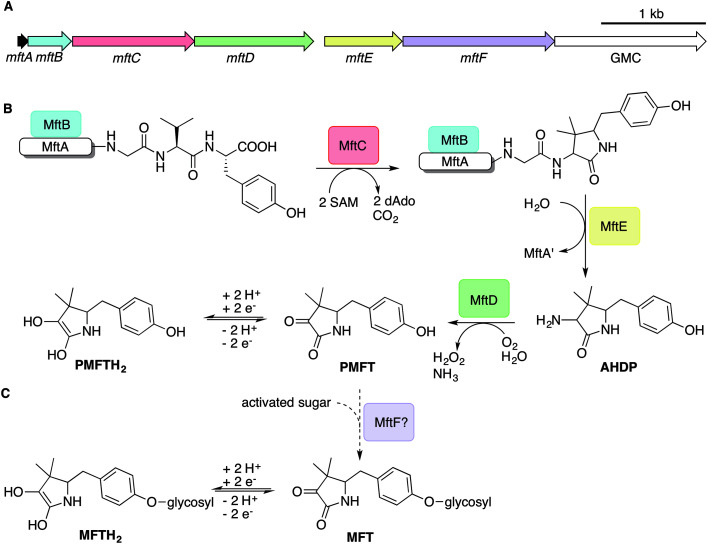
Biosynthesis of mycofactocin. (A) Schematic representation of the MFT biosynthetic gene cluster of *M. smegmatis*. Arrows present genes *mftA-F*. The scale bar indicates 1000 base pairs. (B) Current biosynthesis model of MFT revealed by *in vitro* studies. The precursor peptide MftA (WP_029104568.1) is bound by its chaperone MftB. The rSAM enzyme MftC catalyzes oxidative decarboxylation and cyclization of the core peptide consisting of a C-terminal Val–Tyr dipeptide. The peptidase MftE releases the cyclized core to form AHDP. MftD performs oxidative deamination of AHDP yielding pre-mycofactocin (PMFT), the presumed redox-active core. (C) The putative glycosyltransferase MftF (WP_011727662.1) was hypothesized to glycosylate premycofactocins. (P)MFT is reduced to (P)MFTH_2_ (mycofactocinol) by oxidoreductases. dAdo: 5′-deoxyadenosine, GMC: glucose-methanol-choline oxidoreductase, SAM: *S*-adenosyl methionine.

The architecture of the MFT gene cluster (Fig. 1A) suggested that the resulting natural product is a ribosomally synthesized and post-translationally modified peptide (RiPP).^14^ Several *in vitro* studies have contributed to a preliminary biosynthetic model of MFT (Fig. 1B): the precursor peptide MftA of *M. smegmatis* consisting of 31 amino acids is produced by the ribosome and bound by its chaperone MftB. Subsequently, the terminal core peptide consisting of Val and Tyr is oxidatively decarboxylated and cyclized by the radical SAM enzyme MftC.^15^–^17^ The resulting cyclic core structure is released by the peptidase MftE^18^ forming 3-amino-5-[(*p*-hydroxyphenyl)methyl]-4,4-dimethyl-2-pyrrolidinone (AHDP).^19^ Just recently, it was shown that MftD, an enzyme homologous to the l-lactate dehydrogenase LldD2 20 catalyzes the oxidative deamination of AHDP to yield pre-mycofactocin (PMFT).^21^ The same study demonstrated by voltammetry that the α-keto amide moiety of PMFT is redox-active and can be reduced to PMFTH_2_ (midpoint potential: –255 mV). Efficient reduction was also achieved by the action of carveol dehydrogenase using carveol as an electron donor *in vitro.*^21^ Therefore, PMFT likely represents the redox-center of MFT, as riboflavin is the redox-active core of FMN and FAD.

Although these current hypotheses are plausible, all these known metabolic intermediates have only been observed *in vitro* and could therefore represent artifacts, making the verification of their relevance *in vivo* urgently desired. Furthermore, additional steps of MFT biosynthesis, the function of the *mftF* gene as well as the chemical structure of natural MFT awaited experimental clarification. In this study, we confirm the current biosynthetic model of MFT *in vivo*, detected several novel oligoglycosylated MFT congeners and elucidated their structure. We show that MFTs are decorated with a β-1,4-glucan chain and provide genetic evidence that glycosylation is performed by the glycosyltransferase MftF. Finally, we show dependence of MFT formation on ethanol and corroborate its cofactor function by activity-based metabolic profiling.

## Results and discussion

### Discovery of mycofactocins by metabolomics

**Fig. 2:**
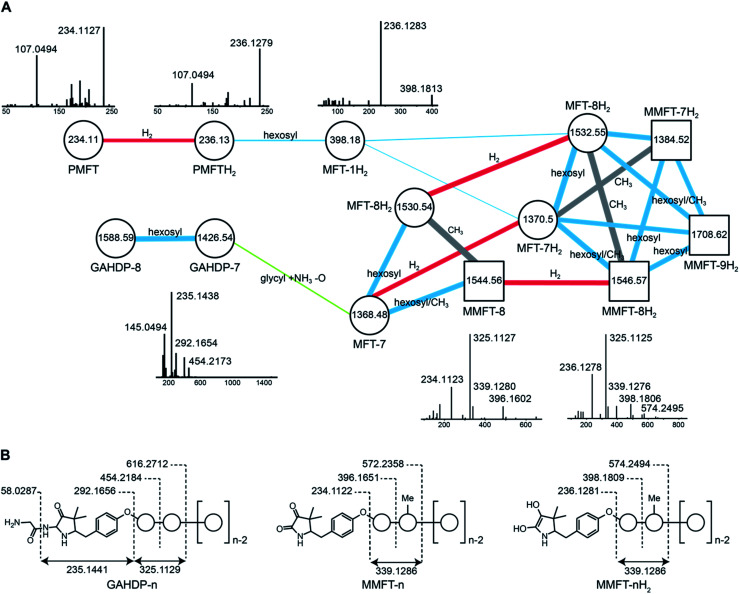
Discovery and tandem mass spectrometry of MFT congeners. (A) Molecular network of MFT congeners. Nodes (circles) represent chemical compounds. Internal node labels display the precursor mass of compounds (*m*/*z* [M + H^+^]). External node labels show proposed compound annotations. Edges represent relationships in terms of shared MS/MS fragments. Edge labels show proposed modifications based on precursor mass shifts (blue: hexosylation, red: oxidation/reduction, grey: methylation). Line widths of edges mirror cosine distances. Representative MS/MS spectra of corresponding precursor ions are shown above or below nodes. (B) Schematic representation of mass fragmentation patterns of GAHDP-*n*, MMFT-*n*H_2_ and MMFT-*n*. Numbers indicate mass-to-charge ratios (*m*/*z*) of fragments observed. Circles represent hexose moieties. Me: methyl group.

In order to identify potential mycofactocin congeners in mycobacteria, we used the fast-growing and weakly pathogenic species *M. smegmatis* MC^2^ 155 as a model organism and developed a metabolomics approach combining metabolic induction and labeling to specifically trace MFT congeners. Assuming that MFT production would be stimulated by alcohols, we cultivated bacteria in media containing 10 g L^–1^ ethanol. Furthermore, we used stable isotope labeling to obtain candidate molecules compatible with the proposed biosynthetic pathway: since the C-terminal core peptide of MftA is composed of Val and Tyr, we reasoned that MFT congeners could be specifically labeled by feeding l-Val-^13^C_5_ and l-Tyr-^13^C_9_. Intracellular contents were extracted and analyzed by liquid chromatography coupled with high-resolution mass spectrometry (LC-MS). Compounds were detected by *in silico* grouping of co-eluting isotopic peaks and adducts (feature finding). Afterwards, ^13^C-labeled compounds were deduced computationally (Data Set 1†). According to the established biosynthetic pathway we expected 13 carbons to remain ^13^C-labeled after oxidative decarboxylation of the Val–Tyr core peptide. We therefore searched for compounds that displayed an exchange of 13 carbons, resulting in a mass shift of +13.04362 Da (ESI Fig. S1†). This approach revealed a list of only twelve candidate compounds. Strikingly, the exact mass and proposed sum formula of three of these compounds corresponded to known intermediates of MFT, recently described *in vitro*, namely AHDP, PMFT as well as PMFTH_2_. In addition to these compounds, several labeled molecules with increasing molecular weight were detected. Some co-eluting candidates with a mass difference of +17.02654 could be explained as NH_4_^+^ adducts of each other. The remaining nine candidate compounds (Table 1) were grouped based on their chromatographic retention times, eluting closely to either PMFT or PMFTH_2_ (approx. 7.2 min and 6.9 min, respectively). Intriguingly, members of the two groups could be arranged in pairs with a mass difference of two hydrogen atoms leading us to assume that each group represented derivatives of either PMFT or PMFTH_2_. Thus, we termed these two clusters of molecules mycofactocinones (MFT) and mycofactocinols (MFTH_2_), respectively. Notably, some mycofactocinols eluted as two chromatographically separated isomers. For instance, the dominant PMFTH_2_ eluted at 6.8 min, while the minor isomer eluted at 6.5 min. These two compounds displayed highly similar MS/MS spectra (ESI Fig. S2†) and most likely represent tautomeric forms. For reasons of simplicity, only the more prevalent isomer was considered during metabolomics studies. We then performed MS/MS networking (Fig. 2), an approach that clusters compounds based on similarity of their MS/MS fragmentation pattern and therefore potentially related chemical scaffolds.^22^

**Table 1:** MFT candidate molecules obtained by stable isotope labeling of *M. smegmatis* with l-Val-^13^C_9_ and l-Tyr-^13^C_9_^*a*^

Name	Sum formula	Exact mass (measured)	RT [min]	Area (mean)
**Aglycons**				
AHDP	C_13_H_18_N_2_O_2_	234.13683	6.53	44 862
PMFTH_2_	C_13_H_17_NO_3_	235.12084	6.88	49 905
PMFT	C_13_H_15_NO_3_	233.10519	7.22	100 832

**Mycofactocinols (MFT-*n*H**_**2**_ **)**				
MFT-1H_2_	C_19_H_27_NO_8_	397.17367	6.88	53 413

**Methylmycofactocinols (MMFT-*n*H**_**2**_ **)**				
MMFT-2H_2_	C_26_H_39_NO_13_	573.24214	6.89	5722
MMFT-7H_2_	C_56_H_89_NO_38_	1383.50626	6.89	340 064
MMFT-8H_2_	C_62_H_99_NO_43_	1545.55908	6.86	550 390

**Methylmycofactocinones (MMFT-*n*)**				
MMFT-7	C_56_H_87_NO_38_	1381.49061	7.25	185 407
MMFT-8	C_62_H_97_NO_43_	1543.54343	7.21	335 701

^*a*^Mycofactocinols (MFT-*n*H_2_) and mycofactocinones (MFT-*n*) represent glycosylated forms of PMFTH_2_ and PMFT, respectively. MMFT-*n*: methylmycofactocinones, MMFT-*n*H_2_: methylmycofactocinols (*n*: number of saccharide moieties). Area values represent the mean of 4 biological replicates. All labeled compounds are shown in Data Set 1, all MFT congeners revealed in this study are shown in Data Set 2.

Interestingly, candidates retrieved from ^13^C-labeling experiments clustered with further putative MFT congeners. The mass difference between the first candidate mycofactocinol (MFT-1H_2_) with an exact mass of 397.17395 Da and PMFTH_2_ was +162.05303 Da, which corresponded to a hexose sugar.

Furthermore, the MS/MS spectrum of MFT-1H_2_ (Fig. 2) showed a fragment ion that corresponded to the mass of the putative aglycon (*m*/*z* 236.13 [M + H]^+^), thus supporting the assumption that MFT-1H_2_ was a glycosylated derivative of PMFTH_2_. MS/MS networking also revealed a recurrent mass difference of 14.01565 between compounds, indicating that methylation might occur as well. We thus assumed that the MFT candidate molecules could be explained as glycosylated or glycosylated and monomethylated species of PMFT(H_2_). In analogy to coenzyme F_420_-*n*, where *n* indicates the number of glutamyl residues in the side chain,^2^ we named the glycosylated molecules MFT-*n*(H_2_) with *n* representing the number of sugar moieties. Monomethylated species were termed methylmycofactocinones (MMFT-*n*) and methylmycofactocinols (MMFT-*n*H_2_), respectively. A targeted search for theoretical mass traces revealed additional members of the MFT-*n*(H_2_) and MMFT-*n*(H_2_) series (Data Set 2†). As expected, the mycofactocinones exhibited MS/MS fragments with a systematic shift by –2.0016 (*e.g.*, *m*/*z* 234.11, 396.16, 572.24) (Fig. 2A) demonstrating that the reduction/oxidation indeed takes place in the PMFT moiety. Oligoglycosylation with up to *n* = 9 saccharide units was detected, while seven and eight units appeared to be the most dominant forms. We observed both methylated (MMFT) and unmethylated (MFT) sugar chains, with the methylated series being more prominent. Only monomethylated species were found. Mass fragmentation of MMFT-*n*(H_2_) species was well in agreement with the assumption that the second sugar was the hotspot for methylation. For instance, MS/MS fragmentation of MMFT-8H_2_ yielded peaks corresponding to ions of MFT-1H_2_ (398.18011) and MMFT-2H_2_ (574.24640) suggesting that the methyl group is present in the second sugar moiety (Fig. 2A).

### Structure elucidation of the oligosaccharide moiety

**Fig. 3:**
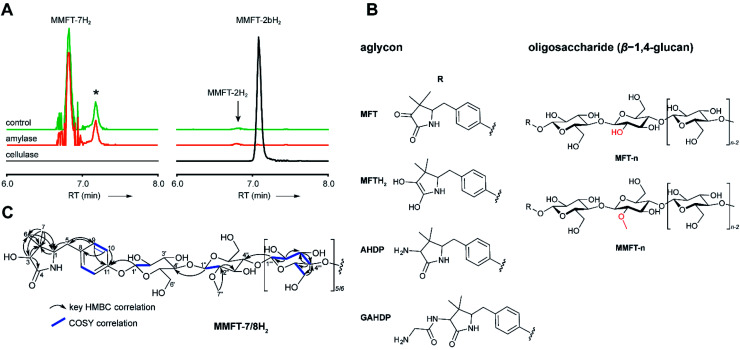
Structure of mycofactocins. (A) Enzymatic degradation of MMFT-*n* by cellulase. Extracted ion chromatograms (XIC, [M + H]^+^) of extract of *M. smegmatis* corresponding to MMFT-7H_2_ (*m*/*z* 1383.50626, left stack) and MMFT-2H_2_ or MMFT-2bH_2_ (*m*/*z* 574.24640, right stack) after treatment with cellulase, amylase, or buffer (control) are shown. Asterisk designates a peak corresponding to the M + 2 isotope of MMFT-7. Digestion by cellulase (β-1,4-glucanase) consumes MMFT-*n*H_2_ and produces MMFT-2bH_2_ suggesting that the oligosaccharide consists of β-1,4-linked glucose. (B) Key COSY and HMBC correlations of MMFT-7/8H_2_. (C) Proposed chemical structures of key mycofactocins and biosynthetic congeners. Mycofactocins are glycosylated by sugar chains consisting of up to nine β-1,4-linked glucose units (*n* ≤ 9). In methylated mycofactocins (MMFT) the second hexose is methylated (2-*O*-methyl-d-glucose). The aglycon is PMFT or PMFTH_2_ in mycofactocinones or mycofactocinols, respectively. The aglycon is AHDP or GAHDP in biosynthetic precursors AHDP-*n* and GAHDP-*n*, respectively.

To determine the structure of elongated mycofactocins, we conducted large-scale cultivations. The dominant mycofactocin exhibited the same mass and fragmentation pattern as MMFT-2H_2_, but eluted at a slightly shifted retention time (Fig. S3†). We therefore named this species MMFT-2bH_2_. Due to the low yields and co-elution of contaminants, structural analysis by nuclear magnetic resonance (NMR) was not possible at this stage. However, cellulase (β-1,4-glucanase) treatment degraded the sugar chain of mycofactocin species (*n* > 2), while amylase (α-1,4-glucanase) did not exhibit any effect (Fig. 3A). This finding strongly suggested that the oligosaccharide chain represents a β-1,4-glucan. Intriguingly, isomer MMFT-2bH_2_, but not MMFT-2H_2_, accumulated after the enzymatic digest, suggesting that MMFT-2b(H_2_) represents the product of cellulase digestion of MMFT-7/8(H_2_) and shares the identical disaccharide anchor. To further elucidate the structure of MMFT-*n*H_2_, we analyzed enriched fractions of MMFT-2bH_2_ and MMFT-*n*H_2_ by chemical derivatization and gas chromatography coupled with mass spectrometry (GC-MS). Monosaccharides were released by acid hydrolysis and derivatized by trimethylsilylation (TMS). Comparative analysis of peaks arising from the MMFT-*n*H_2_ and MMFT-2bH_2_ fractions and carbohydrate standards confirmed the presence of d-glucose (Fig. S4†) and revealed that the methylated sugar present in MMFT-*n*(H_2_) is 2-*O*-methyl-d-glucose (Fig. S5†). To confirm the glycosidic linkage positions, the oligosaccharide was permethylated before hydrolysis so that only hydroxyl groups involved in glycosidic bond formation would be free for silylation.^23^ This experiment (Fig. S6†) lead to the formation of glucose with 2,3,6-*O*-methyl-1,4-*O*-TMS modification confirming the 1,4-glycosidic linkage. Additional modification experiments (methanolysis and permethylation) supported the assignments (Fig. S7–S19†). After repeated cultivation we finally obtained MMFT-7/8H_2_ in sufficient amounts to record 1D and 2D-NMR spectra (ESI results and discussion, Fig. S20–S27, Tables S1 and S2†). The ^1^H NMR spectrum of MMFT-7/8H_2_ exhibited a similar five-membered lactam moiety as present in AHDP, but an isolated methine group was shifted to low-field (*δ*_H-3_ 4.28 ppm/*δ*_C-3_ 76.16 ppm) compared to AHDP (*δ*_H-3_ 3.30 ppm/*δ*_C-3_ 61.64 ppm). This indicated the amine group connected to C-3 was replaced by a hydroxyl group. The HMBC correlation between H-1′ to C-11 suggested the sugar chain to be attached to the hydroxyl group of the tyrosine moiety (Fig. 3B). The β-1,4-glycosidic linkage was confirmed by the HMBC correlations of H-1′′ to C-4′ and H-1′′′ to C-4′′ and the configuration of the glucose moiety was assigned as β-form by the large coupling constant of anomeric protons (*J*_H-1′–H-2′_ = 8.0 Hz; *J*_H-1′′–H-2′′_ = 8.0 Hz; *J*_H-1′′′–H-2′′′_ = 8.0 Hz). The position of the methylated glucose was determined by the observation of a methoxy moiety (*δ*_H-7′′_ 3.64 ppm/*δ*_C-7′′_ 60.55 ppm) and HMBC correlation of H-7′′ to C-2′′. The planar structure of MMFT-7/8H_2_ is presented in Fig. 3C.

In summary, we propose that the oligosaccharide moiety of MFT is a β-1,4-glucane (cellulose). The methylated hexose present in MMFT-*n*(H_2_) and MMFT-2b(H_2_) was shown to be 2-*O*-methyl-glucose. The fact that MMFT-2 and MMFT-2b (digested MMFT-*n*) are distinct in retention times points to some degree of structural diversity within MMFTs. Notably, cellulose was shown to be produced by *M. tuberculosis* as a constituent of biofilms after exposure to reductive stress.^24^ The production of methylated glucans, like 6-*O*-methylglucose lipopolysaccharides (MGPL), albeit with α-1,4 linkage, is well described in *Mycobacteria*, 2-*O*-methylglucose appears to be less common.^25^ Glycosylation is a relatively uncommon modification of cofactors. The most important examples are mycothiol^4^ and bacillithiol.^26^

### Glycine-derived intermediates of MFT biosynthesis

**Fig. 4:**
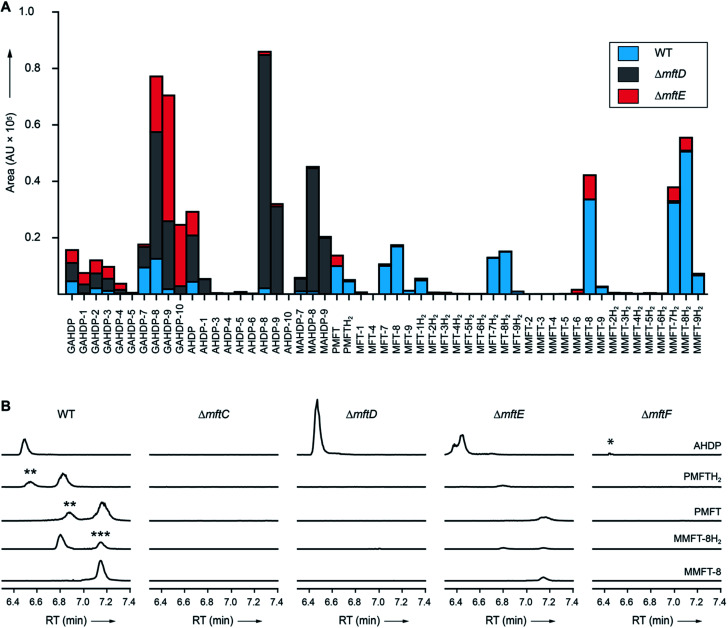
Metabolic profile of MFT congeners present in *M. smegmatis*. (A) Distribution of proposed MFT congeners as determined by LC-MS (Data Set 2†). Bars indicate area under the curve of designated species (average of three biological replicates, *n* = 3). Blue: WT, red: Δ*mftE*, gray: Δ*mftD*. The Δ*mftE* mutant produces significantly reduced amounts of MFT congeners compared to WT, but accumulates incorrectly cleaved products (GAHDP-*n* series). Δ*mftD* is unable to produce PMFT(H_2_) and glycosylated (M)MFT-*n*(H_2_), thus accumulating AHDP-*n* congeners. (B) Extracted ion chromatograms (XIC, [M + H]^+^) of WT and mutants (Δ*mftC*, Δ*mftD*, Δ*mftE*, Δ*mftF*) corresponding to AHDP (*m*/*z* 235.14411), PMFT (*m*/*z* 234.11247), PMFTH_2_ (*m*/*z* 236.12812), MMFT-8 (*m*/*z* 1544.55072) and MMFT-8H_2_ (*m*/*z* 1546.56637). **marks minor isomeric forms (Fig. S2†). ***marks a peak corresponding to the M + 2 isotope of MMFT-8. Δ*mftC* is blocked in biosynthesis of all MFT intermediates, Δ*mftF* abolishes most of the MFT products, but forms trace amounts of AHDP (*). Δ*mftE* produces most MFT species in lower amounts, while intermediates like AHDP are increasing. Δ*mftD* strongly accumulates AHDP, while MFT congeners are abolished.

Surprisingly, the MS/MS network (Fig. 2A) revealed two additional compounds (*m*/*z* 1426.54 and 1588.59) with an unusual mass shift compared to the MFT-*n*(H_2_) candidates. Their mass differences and MS/MS spectra indicated that they represented hepta- and octaglycosylated species sharing a head moiety closely related to PMFT and PMFT(H_2_). The molecular masses and MS/MS spectra of the compounds could be explained by the assumption that the aglycon corresponded to glycyl-AHDP (GAHDP) and these compounds represented the oligoglycosylated forms GAHDP-7 and GAHDP-8. Since the VY core peptide of MftA is preceded by a glycine residue at its N-terminal side it appeared highly likely that the GAHDP-*n* species corresponded to premature cleavage products of the MftC-processed precursor peptide. To corroborate this hypothesis, we fed *M. smegmatis* cultures with a combination of fully ^13^C-labeled Gly-^13^C_2_, Val-^13^C_5_, and Tyr-^13^C_9_. Indeed, GAHDP-derived molecules underwent a mass shift of +15.05033 Da, indicating the incorporation of Gly-^13^C_2_ (+2.00671 Da) in addition to the decarboxylated Val–Tyr moiety (+13.04362 Da) (Fig. S28†). Targeted searches for GAHDP-*n* as well as AHDP-*n* (lacking the glycyl residue) and MAHDP-*n* candidates (AHDP decorated with monomethylated oligosaccharide) revealed three series of oligoglycosylated compounds with similar retention times within each series (Fig. 4, Data Set 2†).

### Dissection of MFT biosynthesis

In order to test if all MFT candidate compounds were related to MFT biosynthesis, we investigated mutants (Δ*mftC*, Δ*mftD*, Δ*mftE*, Δ*mftF*) created previously^12^ of the MFT biosynthesis pathway for the production of candidate molecules (Fig. 4A, Data Set 2†). Indeed, none of the aglycons, nor any of the glycosylated candidates were detected in the Δ*mftC* strain (Fig. 4B). This finding, together with the fact that the genetically complemented strain Δ*mftC*-Comp restored production of MFT congeners (Data Set 2†) represented strong evidence that we indeed identified *bona-fide* MFT-derivatives.

The Δ*mftE* mutant was able to produce mycofactocins, albeit in significantly lower amounts, explaining the previously unexpected phenotypic observation that the Δ*mftE* mutant was able to grow on ethanol, but slower than WT.^12^ Intriguingly, the pool of GAHDP-*n* was strongly increased in the Δ*mftE* strain (Fig. 4A). We thus conclude that MftE can be complemented by an unknown peptidase present in the metabolic background of mycobacteria. Theoretically, an aminopeptidase would be sufficient to degrade the N-terminus of MftA, releasing the AHDP-like core. Peptidases encoded outside the biosynthetic gene cluster have been observed in other RiPP biosyntheses as well.^27^

However, the removal of the glycine residue might be an apparent bottleneck of the alternative maturation pathway in *M. smegmatis*. Alternatively, GAHDPs could represent shunt products that cannot be further processed. In full agreement with the *in vitro* finding that MftD consumes AHDP to form PMFTH_2_,^21^ all metabolites downstream of (M)AHDP-*n* were abrogated in the Δ*mftD* strain, whereas AHDP-*n* and GAHDP-*n* accumulated (Fig. 4). The fact that GAHDP-*n* increased might suggest that the MftE step is impeded in the absence of MftD as well. Genetic dysregulation or cooperative effects between the two enzymes, like complex formation and substrate channeling, might account for this result.

### Glycosylation of MFT is mediated by MftF

It has been speculated that the putative glycosyltransferase MftF catalyzes a final glycosylation of PMFT (Fig. 1C) to yield the mature cofactor.^21^ Our results at this point showed that multiple glucose residues are indeed attached to the aglycon *in vivo*. However, glycosylation appeared already at an early stage as mirrored by the presence of the glycosylated (G)AHDP-*n* series. In order to link oligoglycosylation to a given gene product, we analyzed the Δ*mftF* mutant for the production of glycosylated MFT congeners. Indeed, all glycosylated MFT congeners were abolished in the Δ*mftF* metabolome. Unexpectedly, Δ*mftF* mutants additionally ceased to produce the aglycons PMFT and PMFTH_2_. MftF did, however, produce trace amounts of AHDP, thus showing that at least residual MftC activity was present in the mutant (Fig. 4B). To exclude polar effects, we complemented Δ*mftF* by re-introduction of the *mftF* gene under control of the *mftA* promotor. The restoration of the full MFT metabolite spectrum (Data Set 2†) excluded polar effects and thus verified that MftF was the glycosyltransferase responsible for oligoglycosylation of MFT congeners. The appearance of glycosylated (G)AHDP species in WT together with the drastic decrease of aglycons in Δ*mftF* can be interpreted in a scenario where either glycosylation or the MftF protein itself are essential for the MftD step to efficiently take place *in vivo*. If missing, the biosynthetic machinery may fail to assemble a functional complex or may be unable to recruit the unglycosylated metabolic precursors. The finding that the *mftF* gene is a conserved constituent of MFT biosynthetic loci among different phyla supports the importance of this modification.^11^

The deduced MftF protein of *M. smegmatis* (MSMEG_1426) consists of 470 amino acids (aa) and belongs to the glycosyltransferase 2 family (GT2) according to PFAM (PF00535) and CAZy searches. These enzymes are known for an inverting mechanism of oligoglycoside formation. This is well in agreement with the proposed β-configuration of the MFT oligosaccharide chain. Sequence alignment (Fig. S29A†) showed a high degree of sequence conservation among mycobacterial species and other actinomycetes (*e.g.*, 92% similarity to MftF of *M. tuberculosis* H37Rv). Prediction of transmembrane domains revealed a single helix spanning residues 324–346 with the N-terminus being located outside of the membrane (Fig. S29B†). The MMFT biosynthetic machinery, however, appears not to be fully encompassed within the MFT cluster since no methyltransferase was found. Future studies are warranted to identify the enzymes involved in MFT oligosaccharide methylation.

### Cofactor role of mycofactocin

**Fig. 5:**
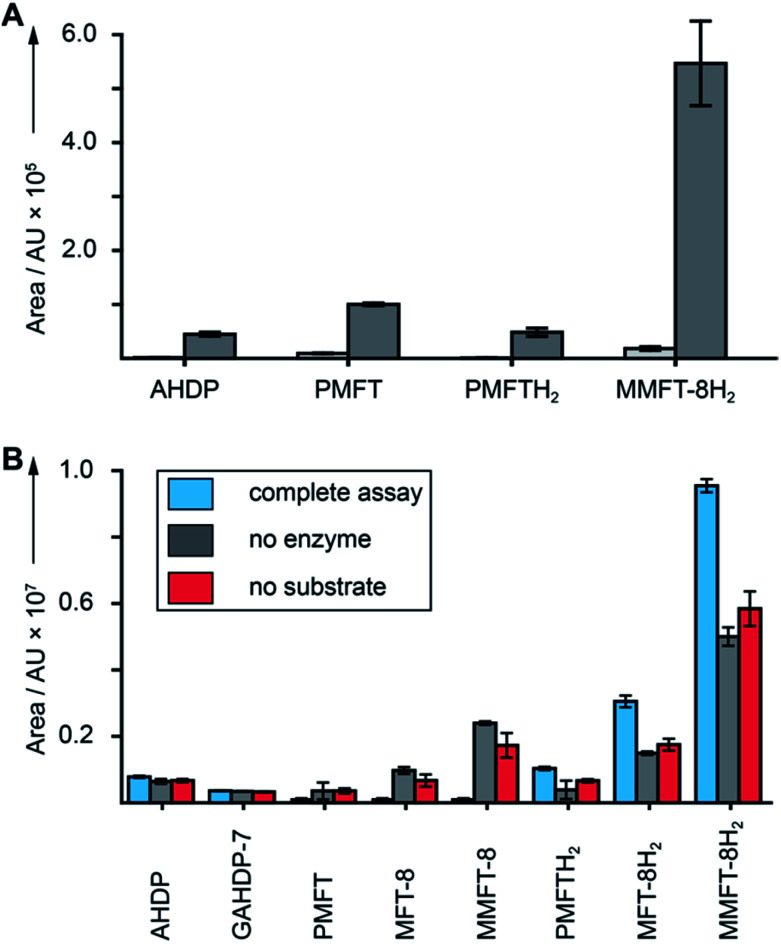
Cofactor role of mycofactocin. (A) MFT congeners are strongly upregulated (MMFT-8H_2_: 26-fold) on ethanol-containing media. Area under the curve of MFT species produced by *M. smegmatis* treated with ethanol (dark gray) *versus* glucose controls (light gray) are shown (Data Set 3†). (B) Reduction of mycofactocinones to mycofactocinols by treatment of extracts with LimC and carveol. Blue: complete assay with enzyme and l-carveol as substrate. Red: control without substrate, gray: control without enzyme. Bars represent average area under the curve, error bars standard deviation of 3 biological replicates (*n* = 3) for in both charts.

After discovery of the glycosylated mycofactocins, we examined to which extent their production was actually dependent on the presence of ethanol in culture media. We therefore systematically compared the metabolome of *M. smegmatis* WT after ethanol treatment with glucose controls (Data Set 3†). The results demonstrated that all MFT congeners or intermediates were strongly upregulated upon cultivation on ethanol (median: 34-fold upregulation) (Fig. 5A). These data perfectly support a recent report that MFT is involved in alcohol metabolism.^12^

Finally, we sought to confirm that the MFT congeners identified in this study are indeed coenzymes of MFT-dependent enzymes. To assess this question, we turned to activity-based metabolic profiling.^28^ We incubated the extracted metabolome of *M. smegmatis* with the recombinant l-carveol dehydrogenase LimC (CAB54559.1) from *Rhodococcus erythropolis* (Fig. S30†), a nicotinoprotein with a non-exchangable NADH cofactor.^29^ This enzyme was proposed to require MFT as an external electron acceptor.^11^ A recent study showed that carveol dehydrogenase from *M. smegmatis* was able to reduce PMFT to PMFTH_2_ using carveol and internally bound NADH as an electron donor.^21^ Likewise, we observed full reduction of all mycofactocinones to mycofactocinols (Fig. 5B, Data Set 4†) by LimC when combined with carveol as a substrate. Controls lacking enzyme or substrate showed weak and no turnover, respectively. The low turnover by LimC alone can be explained by internally bound NADH as reported before.^21^ Both the aglycon PMFT as well as the oligoglycosylated MFT-*n* and MMFT-*n* species were completely turned over, while redox-inactive AHDP congeners remained unaffected. These data further validate the notion that all MFT candidates presented here are mycofactocins with full cofactor function. It remains to be clarified if there is a preference for the glycosylated coenzymes or their aglycons in the bacterial cell.

## Conclusion

The redox cofactor mycofactocin has attracted considerable interest since it was postulated by bioinformatics. Despite recent progress made by *in vitro* studies, evidence for mycofactocin congeners in living microorganisms has been missing so far. Our integrated metabolomics approach combining stable isotope labeling, metabolite induction, MS/MS networking as well as genetic dissection of the biosynthetic pathway turned out to be a powerful approach to identify RiPP congeners in bacteria and could inspire similar projects in the future. Using this technique, we discovered natural MFT and found that it is decorated with oligosaccharides consisting of up to nine β-1,4-linked glucose units. Analyses of Δ*mftF* mutants and complement strains revealed that MftF is the glycosyltransferase responsible for the oligoglycosylation observed. Mycofactocins can be isolated in oxidized (mycofactocinones) and reduced forms (mycofactocinols) and are co-substrates of enzymatic reduction by carveol dehydrogenase. These data provide strong evidence that mycofactocins are indeed redox cofactors as proposed earlier.^11^,^12^,^21^ We, therefore, conclude that we have finally discovered the family of compounds that was tentatively called “mycofactocin” and thus close an important gap of knowledge in the field. Our results will guide further studies into the occurrence, physiological role, and biochemistry of mycofactocins in microorganisms. Finally, these and other studies will inspire future efforts to exploit mycofactocin, *e.g.*, as a disease marker or as a potential drug target for the treatment mycobacterial infections.

## Conflicts of interest

There are no conflicts to declare.

## Supplementary Material

Supplementary information

Supplementary information

Supplementary information

Supplementary information

Supplementary information
