# Supplementary material for: Structure elucidation of the redox cofactor mycofactocin reveals oligo-glycosylation by MftF
Source: Chem Sci. 2020 Apr 23;11(20):5182–90. doi: 10.1039/d0sc01172j (PMC7491314; doi:10.1039/d0sc01172j)
Supplement: Supplementary file 5 [file SC-011-D0SC01172J-s005.pdf]

Electronic Supplementary Material (ESI) for Chemical Science.
This journal is © The Royal Society of Chemistry 2020

Electronic Supplementary Information - Appendix

Structure elucidation of the redox cofactor mycofactocin reveals oligo-glycosylation by MftF.

Luis Peña-Ortiz[1,2], Ana Patrícia Graça[1,2], Huijuan Guo[3], Daniel Braga[1,2], Tobias G. Köllner[4], Lars Regestein[5], Christine Beemelmanns[3], and Gerald Lackner[1,2*]

AUTHOR AFFILIATIONS

[1]Junior Research Group Synthetic Microbiology
Leibniz Institute for Natural Product Research and Infection Biology (HKI)
Beutenbergstr. 11a, 07745 Jena, Germany

[2]Friedrich Schiller University
Beutenbergstr. 11a, 07745 Jena, Germany

[3]Junior Research Group Chemical Biology of Microbe-Host Interactions
Leibniz Institute for Natural Product Research and Infection Biology (HKI)
Beutenbergstr. 11a, 07745 Jena, Germany

[4]Department of Biochemistry
Max Planck Institute for Chemical Ecology
Hans-Knöll-Str. 8, 07745 Jena, Germany

[5]Bio Pilot Plant
Leibniz Institute for Natural Product Research and Infection Biology (HKI)
Beutenbergstr. 11a, 07745 Jena, Germany

*** Corresponding Author:**

Dr. Gerald Lackner

Junior Research Group Synthetic Microbiology
Leibniz Institute for Natural Product Research and Infection Biology (HKI)
Beutenbergstr. 11a, 07745 Jena, Germany

E-mail: gerald.lackner@leibniz-hki.de

Phone: +49 3641 532 1104

ABSTRACT

Mycofactocin (MFT) is a redox cofactor belonging to the family of ribosomally synthesized and post-translationally modified peptides (RiPPs) and is involved in alcohol metabolism of mycobacteria including *Mycobacterium tuberculosis*. A preliminary biosynthetic model had been established by bioinformatics and *in-vitro* studies, while the structure of natural MFT and key biosynthetic steps remained elusive. Here, we report the discovery of glycosylated MFT by ^{13}C-labeling metabolomics and establish a model of its biosynthesis in *Mycolicibacterium smegmatis*. Extensive structure elucidation including NMR revealed that MFT is decorated with up to nine β-1,4-linked glucose residues including 2-*O*-methylglucose. Dissection of biosynthetic genes demonstrated that the oligoglycosylation is catalyzed by the glycosyltransferase MftF. Furthermore, we confirm the redox cofactor function of glycosylated MFTs by activity-based metabolic profiling using the carveol dehydrogenase LimC and show that the MFT pool expands during cultivation on ethanol. Our results will guide future studies into the biochemical functions and physiological roles of MFT in bacteria.

Table of Contents

Experimental **3**

Microbial strains and general cultivation conditions 3

Generation *of M. smegmatis* mutant and complement strains 3

Isotopic labeling of M. smegmatis 3

Comparative metabolomics studies 4

Metabolite extraction and LC-MS measurements 4

MS/MS networking 4

Bioinformatics analysis of the MftF primary structure 5

Heterologous production and purification of LimC 5

Activity-based metabolic profiling 5

Glucanase digestion of mycofactocins 6

Enrichment of the MMFT-2b(H_2) 6

Enrichment of MMFT-n 7

Determination of the sugar composition 7

Permethylation 7

Acid hydrolysis 7

Methanolysis 7

Silylation and GC-MS 7

Gas chromatography - mass spectrometry (GC-MS) 8

Purification of MMFT-7/8-H_2 8

Nuclear magnetic resonance (NMR) spectroscopy 8

Results and Discussion **9**

Discovery of mycofactocins 9

GC-MS analyses of the oligosaccharide moiety 12

Structure elucidation of MMFT-7/8-H_2 by NMR 28

Additional results 35

Plasmid Sequences **38**

References **43**

Experimental

Microbial strains and general cultivation conditions

The *M. smegmatis* MC2 155 WT and mutant strains ΔmftC, ΔmftD, ΔmftE and ΔmftF were maintained in lysogeny broth (LB), supplemented with 0.05% Tween80 (LB-Tween) at 37 °C and 180 rpm. Complement mutants *M. smegmatis mftC*-Comp and *mftF*-Comp were maintained in media containing kanamycin (20 µg mL-1*). Escherichia coli* TOP10 was grown in LB supplemented with kanamycin (30 µg mL^{-1}) at 37 °C for the generation and maintenance of pMCpAINT-derived plasmids. Metabolomics studies were carried in adapted HdB medium(Berney, Weimar et al., 2012) containing 3 g L^{-1} Na_2HPO_4. Liquid cultures were supplemented with 0.5 g L^{-1} tyloxapol, 10 g L^{-1} glucose (w/v) or 10 g L^{-1} ethanol (w/v) at 37 °C and 180 rpm.

Generation *of M. smegmatis* mutant and complement strains

Scarless mutants of biosynthetic genes of *M. smegmatis* (*ΔmftC, ΔmftD ΔmftE*, *ΔmftF)* as well as the *mftC* complement strain (Δ*mftC*-Comp) were obtained from the Kaufmann laboratory(Krishnamoorthy, Kaiser et al., 2019). For the genetic complementation of *M. smegmatis* Δ*mftF*, the promotor and the ribosome binding site of *mftA* were combined with the *mftF* coding sequence (obtained as a synthetic DNA construct) and cloned as an insert of the integrative plasmid pMCpAINT(Warner, Ndwandwe et al., 2010) yielding plasmid pPG20 (Figure S31). After transformation of electrocompetent cells of *M. smegmatis* by electroporation, positive clones (Δ*mftF*-Comp) were selected on kanamycin (20 µg mL^{-1}). Complement mutants were confirmed phenotypically by the (restored) ability to grow in HdB supplemented with 10 g L^{-1} ethanol as a sole source of carbon as well as genetically by positive PCR amplification of the *mftF* gene using the following primers: INT_mftF_F: 5'-ACTTCTCCGGTATGCACTGC-3' and INT_mftF_R1: 5'-ACAGATCGCCGAACACAACT-3').

Isotopic labeling of *M. smegmatis*

A saturated pre-culture of *M. smegmatis* MC^2 155 WT in LB broth was used to inoculate 25 mL of HdB medium supplemented with 0.5 g L^{-1} tyloxapol and 10 g L^{-1} ethanol to an initial optical density at 600 nm (OD_{600}) of 0.1. Cultivations contained 1 mM L-tyrosine-$^{13}C_9$ (99% atom purity, Cortecnet) and 1 mM L-valine-$^{13}C_5$ (99% atom purity, Merck) and were conducted in four replicates at 37 °C and 180 rpm for 24 h. Cultures (10 mL of a 10-fold dilution) were poured onto sterile regenerated cellulose filters (0.2 µm, Sartorius) previously conditioned with water. The biomass was repeatedly washed with sterile water and transferred to HdB agar plates with 10 g L^{-1} ethanol and with either light or heavy L-valine and L-tyrosine supplementation, as appropriate. Filters inoculated with sterile media were used as control. Directly after the incubation period (37 °C for 48 h), the filters were extracted and subjected to LC-MS measurements as described below. Data analysis was performed with the Stable Isotope Labeling workflow of Compound Discoverer 3.0 (Thermo Scientific) allowing for a maximum exchange of 16 ^{13}C atoms. Independent analyses were performed for the lower and higher scan ranges. A minimal peak intensity cut-off of 10^4 was defined for compound detection. Compounds with the same mass (± 5 ppm) and eluting within 0.2 min from each other were grouped. Molecules were considered candidates potentially comprising the decarboxylated Val-Tyr core peptide (*i.e.* AHDP moiety) if a relative ^{13}C-exchange rate higher than 50% was observed. Low abundance compounds (area <1000) and candidates containing a high proportion of contaminating masses were disregarded.

Comparative metabolomics studies

Cultures of *M. smegmatis* MC^2 155 WT as well as Δ*mftC*, Δ*mftD*, Δ*mftE*, Δ*mftF*, Δ*mftC*-Comp and Δ*mftF*-Comp growing in LB supplemented with 0.05% of Tween80 were used to inoculate sterile regenerated cellulose filters as described before, standardizing all the cultures to the same concentration. The filters were incubated in HdB supplemented with 10 g L^{-1} glucose at 37 °C for 18h. Afterwards, the filters were transferred to a new HdB plate supplemented either with 10 g L^{-1} of glucose or 10 g L^{-1} ethanol and incubated at 37°C for 18h. This study was carried out in triplicates and filters incubated with media were used as control (blank). Directly after the incubation period, the filters were further extracted and subjected to LC-MS measurements as described below. Targeted studies were performed using the Expected Compounds node in Compound Discoverer 3 with an Expected Compounds table including AHDP, PMFT, $PMTH_2$, MFT-1 and MFT-1H2 allowing for multiple glycosylation and methylation events. Compounds from different runs with the same mass (< 5 ppm deviation) and eluting within 0.2 min from each other were grouped. Median of areas under the curve of three replicates was used to compute ratios between groups.

Metabolite extraction and LC-MS measurements

Filters were recovered with sterile tweezers and placed on 20 mL chilled extraction mixture (acetonitrile:methanol:water 60:20:20 with 0.1% formic acid). Bottles were placed one hour at -80 °C, sonicated for 5 min in an ultrasonic bath at room temperature and frozen. The lysis procedure was repeated three times. After the last ultrasound treatment, extract was transferred to round flasks, frozen and lyophilized. The dry extract was resuspended in 950 µL ddH_2O, centrifuged twice and the final extract was saved in HPLC vials at -20 °C until measurement. LC-MS measurements were performed on a Dionex Ultimate 3000 system combined with a Q Exactive Plus mass spectrometer (Thermo Scientific) equipped with a heated electrospray ion source (HESI). Metabolite separation was carried out using a Phenomenex Kinetex XB-C18 column (150 × 2.1 mm, 2.6 µm, 100 Å) preceded by a Phenomenex SecurityGuard ULTRA guard cartridge (2 × 2.1 mm). Mobile phases consisted of 0.1% formic acid in either water (A) or acetonitrile (B). 10 µl of the sample were separated chromatographically at 40 °C and a constant flow rate of 300 µL min^{-1} as follows: 0-2 min, 2% B; 2-15 min 2-99% B; 15-18 min 99% B. Metabolite separation was followed by both full scan (MS^1) and data-dependent MS/MS (MS^1 and Top10 MS/MS) analyses in positive ionization mode at two scan ranges: *m/z* 200 to 600 and *m/z* 580 to 2000. Spray quality was adjusted based on the ratio of water:acetonitrile. For the first 10 min of elution sheath gas flow rate was set to 35 and auxiliary gas flow rate to 7. For the following 12 min those values were set to 24 and 2, respectively. Capillary temperature was 320 °C, probe heater temperature was 230 °C, spray voltage was 4 kV, and S-lens RF level was 50 at all times. MS^1 had the resolving power set to 70,000 FWHM at *m/z* 200, injection time to 100 ms, and AGC to 3E6. The ten most intense ions were selected for MS/MS with a scan rate of 12 Hz with a dynamic exclusion of 10 s. Resolving power was 17,500 FWHM at *m/z* 200, AGC target was 1E5, and injection time was 50 ms. Isolation window was set to *m/z* 1, while routine analysis was performed at 40 NCE (normalized collision energy). Targeted MS/MS spectra acquisition for the MFT congeners was performed at NCE values of 20, 30, and 40.

MS/MS networking

M. smegmatis MC2 155 metabolome extracts were prepared as described before. Data-dependent (TOP 10) MS/MS data was acquired and converted to the mzXML format with the MSConvert tool(Chambers, Maclean et

al., 2012). Files were uploaded to the Global Natural Products Social Molecular Networking (GNPS) server(WangCarver et al., 2016) and analyzed with the Molecular Networking pipeline. Parameters were set as default except for the following: minimum cosine score (0.6) and minimum fragment ions (4). Results were visualized in Cytoscape 3.7(Shannon, Markiel et al., 2003).

Bioinformatics analysis of the MftF primary structure

The primary protein structure of several MftF homologues was downloaded from the NCBI database. Sequences were aligned using the MUSCLE algorithm(Edgar, 2004) implemented in Geneious Prime (2019.1.1). Prediction of transmembrane domains was performed using the TMHMM 2.0 webserver(Krogh, Larsson et al., 2001). Classification of MftF was performed by the carbohydrate-active enzymes database (CAZy)(Lombard, Golaconda Ramulu et al., 2014).

Heterologous production and purification of LimC

The *limC* gene encoding the carveol dehydrogenase LimC(van der Werf, van der Ven et al., 1999) from *Rhodococcus erythropolis* DCL14 was obtained as a codon optimized synthetic construct inserted in vector pET28, in-frame with the N-terminal hexahistine tag (plasmid pLAPO4, see Figure S31a). A single colony of *E. coli* BL21(DE3) freshly transformed with plasmid pLAPO4 was inoculated in 5 mL LB with kanamycin (50 µg mL^{-1}) and cultured overnight at 37 °C and 210 rpm. A main culture (100 mL) was inoculated in LB media amended with the same antibiotic and cultured until an OD_{600} of 0.5 – 0.6. At this point expression of *limC* was induced with IPTG (0.5 mM) and the temperature decreased to 16 °C, then further cultured for 18 – 20 h. Cells were harvested from the medium by centrifugation at 4000 × g and 4 °C for 30 min, then resuspended and washed in cold sodium chloride 0.85% and harvested again for 15 min, then frozen at -20 °C for future use or processed as follows. The cell pellet was resuspended in lysis buffer (50 mm NaH_2PO_4, 300 mm NaCl, pH 8.2) containing 20 mM imidazole, disrupted with a Sonopuls ultrasonic sonifier (Bandelin), and centrifuged at 17000 × g, 4 °C for 30 min. The lysate was applied to 1.25 mL HisPur NiNTA resin (Thermo Fisher Scientific), pre-equilibrated with 10 column volumes of lysis buffer at the same imidazole concentration and allowed to flow at room temperature by gravity. The resin was washed with 10 CV of wash buffer (lysis buffer containing 30, 50, and 70 mM imidazole). The target protein was then eluted with 2.5 mL of lysis buffer containing 300 mM imidazole, then immediately rebuffered in 3.5 mL assay buffer (sodium citrate buffer 50 mM, pH 6) in a PD-10 gel filtration column (GE Healthcare) and concentrated to a final volume of 500 µL in a 3 kDa MWCO Vivaspin centrifugal filtration unit (Sartorius). The protein was detected by SDS-PAGE (Figure S30), protein concentration was determined with the Roti Nanoquant (Carl Roth) reagent following manufacturer instructions in relation to known concentrations of BSA in assay buffer. Positive enzymatic activity was confirmed by incubating with L-carveol (1 mM) in the presence of 0.1 mM DCPIP. Discoloration in the presence of carveol indicated positive activity.

Activity-based metabolic profiling

Metabolome extracts of *M. smegmatis* MC^2 155 were prepared as described above and used for activity-based metabolic profiling. Metabolome extract (10 µL of a 10-fold dilution) was mixed with 0.1 µg of purified LimC in assay

buffer (sodium citrate buffer 50 mM, pH 6) containing L-carveol (1 mM) in a 20 µL reaction volume. Negative controls were performed without substrate or without enzyme. The reaction was quenched after 1 h of incubation at room temperature by adding 1 volume of LC-MS grade acetonitrile and centrifugation for 10 min at 4°C. 10 µL were injected for LC-MS measurement as described below. Mycofactocinone and mycofactocinol species were identified using Compound Discoverer 3.1 based on *m/z* and retention time.

Glucanase digestion of mycofactocins

Metabolome extracts of *M. smegmatis* MC^2 155 were prepared as described above and mixed with 0.2 mU of commercial cellulase (1,4-β-glucanase) from *Trichoderma reesii* ATCC 26921 (Merck) in 50 mM citrate-phosphate buffer at pH 5 (6.5 mM citric acid, 43.6 mM sodium phosphate) or 0.2 mU α-amylase (1,4-α-glucanase) from *Bacillus licheniformis* (Merck) in citrate-phosphate buffer at pH 7 (24.3 mM citric acid, 25.7 mM sodium phosphate). Enzyme solutions were prepared at a concentration of 0.2 mU µL^{-1}. 30 µL reactions were set up with 1 µL enzyme, 14 µL of metabolome extract and 15 µL of concentrated buffer (2 $\times$). Buffer replaced the enzyme solution at pH 5 or 7 in control measurements. The reaction was incubated for 1 hour at the optimum temperature for each enzyme, then quenched by the addition of 1 volume acetonitrile (LC-MS grade) and centrifuged at 12000 $\times$ g for 10 min. 5 µL of the supernatant were injected for LC-MS measurements.

Enrichment of the MMFT-2b(H_2)

The first pre-culture of *M. smegmatis* WT was grown for 18 h in serum bottles under the following conditions: shaking frequency of 180 rpm, shaking diameter of 25 mm and a flask volume of 50 ml with 20 ml filling volume. The second pre-culture step was conducted in Erlenmeyer shake flaks for 18 h under the following cultivation conditions: shaking frequency of 180 rpm, shaking diameter of 25 mm and a flask volume of 2 L with a filling volume of 400 mL. The transferred volume of the second pre-culture was 1 L to inoculate a 75 L stirred tank reactor filled with 50 L LB medium supplemented with 20 g L^{-1} ethanol and 20 g L^{-1} glucose for 23.25 h of cultivation. To ensure aerobic conditions, the dissolved oxygen tension was controlled by the stirring rate and therefore always higher than 20%. The gas flow rate was constant at 0.5 vvm as well as the headspace overpressure of 0.2 bar. For biomass separation, the overall fermentation broth was filtered and the received cells were extracted twice with MeOH. The MeOH extract was separated from the cell debris by filtration and concentrated under reduced pressure. LC-MS analysis of crude MeOH extract revealed the presence of MMFT-2bH_2 with the major detectable mycofactocin species, with the observed molecular ion at *m/z* 574.24945 ($[M+H]^+$), and MMFT-2b in trace amounts, with the observed molecular ion at *m*/*z* 572.23431 ($[M+H]^+$). The concentrated MeOH extract was first suspended into 10% MeOH (50 mL) by ultrasonication, then fractionated by SPE-C18 cartridge (10 g) with the step elution by 10% MeOH, 20% MeOH, 30% MeOH, 40% MeOH, 50% MeOH, 60% MeOH, 80% MeOH, and pure MeOH, and concentrated under reduced pressure. LC-MS analysis of individual fractions indicated that the MMFT-2bH_2 was accumulated in 30% MeOH fraction, with a small amount in 40% – 60% MeOH. The 30% MeOH fraction, as well as 40% – 60% MeOH fractions were submitted to size exclusion by Sephadex LH20 eluted with 50% MeOH, and fractionated into six fractions. Following the LC-MS analysis of each fraction, the MMFT-2H_2 molecular ion was located in the third fraction (Fr.3-3). In the end the Fr.3-3 was submitted to semiprepative HPLC coupled with Phenomenex Luna C8 column (100 Å 250 $\times$ 10 mm) and separated under the gradient of 0 – 5 min, 10% MeOH; 5 – 20 min, 10% – 60% MeOH; 20 – 25 min, 60% MeOH; 25 – 35 min, 60% – 100% MeOH; 35 – 40 min, 100%

MeOH, with the flow rate of 2.0 mL min^{-1}. LC-MS indicated that MMFT-2bH_2 was enriched in the fraction with t_R = 22.6 min, and the mixture of MMFT-2bH_2 and MMFT-2b was enriched in next fraction with t_R = 23.0 min.

Enrichment of MMFT-n

For large-scale cultivation, pre-cultures of *M. smegmatis* WT in LB-Tween were used (1:200) for inoculation of batches of 400 mL in 1 L flasks of LB-Tween supplemented with 10 g L^{-1} ethanol. In total, 24 L culture broth was inoculated, centrifuged, and bacterial pellet was collected and frozen at –80 °C before extraction in batches. 200 mL of MeOH was added into a glass flask containing thawed bacteria and stirred at 4 °C overnight. The yellowish MeOH extract was filtrated and concentrated under reduced pressure, suspended into 10% MeOH by ultrasonication and finally loaded on a SPE C18 cartridge (5 g). The fractionation was performed by step elution with 10% MeOH, 20%, 30%, 40%, 50%, 60%, 80%, 100% MeOH. LC-MS analysis of individual fractions indicated that the MMFT-n was accumulated in 30% MeOH and 40% MeOH fractions. These two fractions were submitted to size exclusion by Sephadex LH20 eluted with 50% MeOH, and fractionated into six fractions, respectively. Based on the LC-MS analysis of each fractions, the MMFT-n(H_2) enriched fraction was further purified by semipreparative HPLC. MMFT-n(H_2) containing fractions (t_R = 22.48 min) were pooled and submitted to sugar composition analysis mediated by chemical degradation, modification and finally by GC-MS analysis.

Determination of the sugar composition

Permethylation

The enriched MMFT-n(H_2) and MMFT-2b(H_2) (50 µg) were transferred into a screw cap glass vial (4 mL), and dried completely in an evaporator (GeneVac). An amount of 0.5 mg of authentic standard cellulose was weighed into a 4 mL screw cap glass vial individually. To each of these samples, 0.5 mL of dried dimethyl sulfoxide (DMSO) was added together with 100 µL of iodomethane. To this solution 60 mg of finely ground NaOH was added in excess. (The pellets of NaOH were ground in a hot and dried mortor and pestle (120 °C oven for 30 min). The reaction was performed on a shaker at 50 °C for 30 min, and quenched by the addition of ice-cold water to prevent a high temperature and degradation. A liquid-liquid extraction using dichloromethane (DCM) and subsequenct washes with ice-cold water were performed three times to remove NaOH and DMSO. The upper aqueous layer was discarded, and the remaining bottom organic layer containing permethylated products was collected and dried to completion.

Acid hydrolysis

50 µg of the enriched MMFT-n(H_2) and MMFT-2b(H_2), or permethylated products were transferred into a 4 mL screw cap glass vial and dried completely by GeneVac. The acid hydrolysis was performed by adding 1 mL of HCl (3 M, aq.) and kept at 95 °C for 5 hours. Afterwards, the whole mixture was dried thoroughly by GeneVac.

Methanolysis

50 µg of the enriched MMFT-n(H_2), MMFT-2b(H_2), or the permethylated products were transferred into 4 mL screw cap glass vial and dried completely by GeneVac. The methanolysis were performed by adding 1 mL of HCl in MeOH (1.25 M) and kept at 95 °C for 5 hours. Afterwards, the whole mixture was dried thoroughly by GeneVac.

Silylation and GC-MS

The released monosaccharide residues were derivatized with MSTFA (MSTFA: *N*-methyl-*N*-(trimethylsilyl)trifluoroacetamide) by adding 25 μL dried pyridine and 25 μL MSTFA and kept at 60 ^{o}C for 30 min. Silylated samples were subjected to analysis by GC-MS coupled with a ZB5 column as described below. The authentic standard monosaccharides (D-(+)-glucose, methyl-α-D-glucose, methyl-β-D-glucose, 2-OMe-D-(+)-glucose, 3-OMe-D-glucose, 4-OMe-D-glucose, and 6-OMe-D-glucose) were purchased from Sigma-Aldrich or Carbosynth, derivatized and analyzed under the same procedure.

Gas chromatography - mass spectrometry (GC-MS)

GC-MS analysis was conducted using an Agilent 6890 Series gas chromatograph coupled to an Agilent 5973 quadrupole mass selective detector (interface temp, 270 °C; quadrupole temp, 150 °C; source temp, 230 °C; electron energy, 70 eV). Compounds were separated using a ZB5 column (Phenomenex, Aschaffenburg, Germany, 30 m × 0.25 mm × 0.25 μm) and He (1.5 ml min^{-1}) as carrier gas. The sample (1 μL) was injected with a split of 50:1 at an initial oven temperature of 100 °C. The temperature was held for 2 min and then increased to 250 °C with a gradient of 7 °C min^{-1}, and then further increased to 330 °C with a gradient of 100 °C min^{-1} and a hold of 3 min. Compounds were identified by comparison of retention times and mass spectra to those of authentic standards or by reference spectra in the Wiley and National Institute of Standards and Technology (NIST) libraries.

Purification of MMFT-7/8-H_2

Cultures of *M. smegmatis* WT were grown in a fermentor as described above. The pellet was re-extracted by 200 mL of 50% MeOH/50% H_2O and 200 mL 40% MeOH/60% H_2O under ultrasonication for 30 min, respectively. The yellow extract was collected by centrifugation under 10,000 g for 10 min at r.t. and concentrated under reduced pressure. The MeOH-H_2O extracts were combined and further separated by SPE-C18 cartridge (5 g) performed by step elution from 10% MeOH, 20%, 30%, 40%, 50%, 60%, 80%, 100% MeOH. HRMS analysis of individual fractions indicated that the MMFT-n was accumulated in 30% MeOH and 40% MeOH fractions, in which the major species were MMFT-7-H_2 and -8-H_2 with dominate reduced form (*m*/*z* 1384.50804 and 1546.55939) and trace oxidative form (*m*/*z* 1382.49301 and 1544.54459). These two fractions (30% MeOH and 40% MeOH) were submitted to purify by semipreparative HPLC coupled with Phenomenex Luna C8 250 x 10 mm with the gradient: 0–5 min, 10% B; 5–20 min, 10%–60% B; 20–25 min, 60% B; 25–35 min, 60%–100% B; 35–40 min, 100% B; 40–42 min, 100%–10% B; 42–50 min, 10% B (A: ddH_2O; B; MeOH) with the flow rate of 2.0 mL/min. Mixture of MMFT-7-H_2 and -8-H_2 fraction (0.88 mg) was collected at t_R = 21.7 min and concentrated under reduced pressure by GeneVac.

Nuclear magnetic resonance (NMR) spectroscopy

NMR measurements were performed on a Bruker AVANCE III 600 MHz spectrometer, equipped with a Bruker Cryoplatform. The chemical shifts are reported in parts per million (ppm) relative to the solvent residual peak of D_2O (^{1}H: 4.79 ppm, singlet).

Results and Discussion

Discovery of mycofactocins

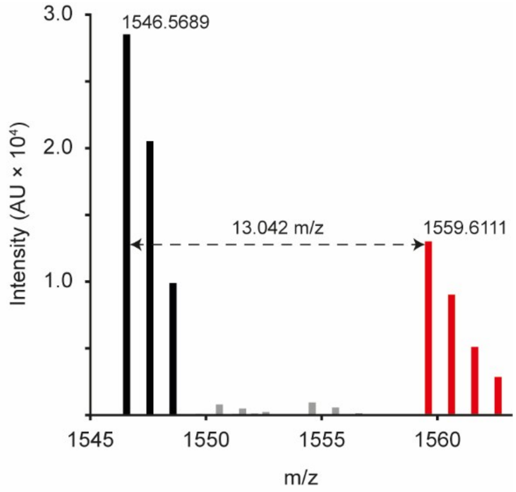

Figure S1. Stable isotope labeling of MFT congeners.

After feeding of L-Val-$^{13}C_5$ and L-Tyr-$^{13}C_9$ the mass spectrum of MMFT-8H_2 (*m/z* 1546.56809, $[M+H]^+$) demonstrated incorporation of 13 ^{13}C labels (expected mass shift: 13.04362 Da). Black: Natural isotope distribution of metabolites. Red: Isotope pattern of labeled metabolite. Figure represents an overlay of two individually recorded mass spectra.

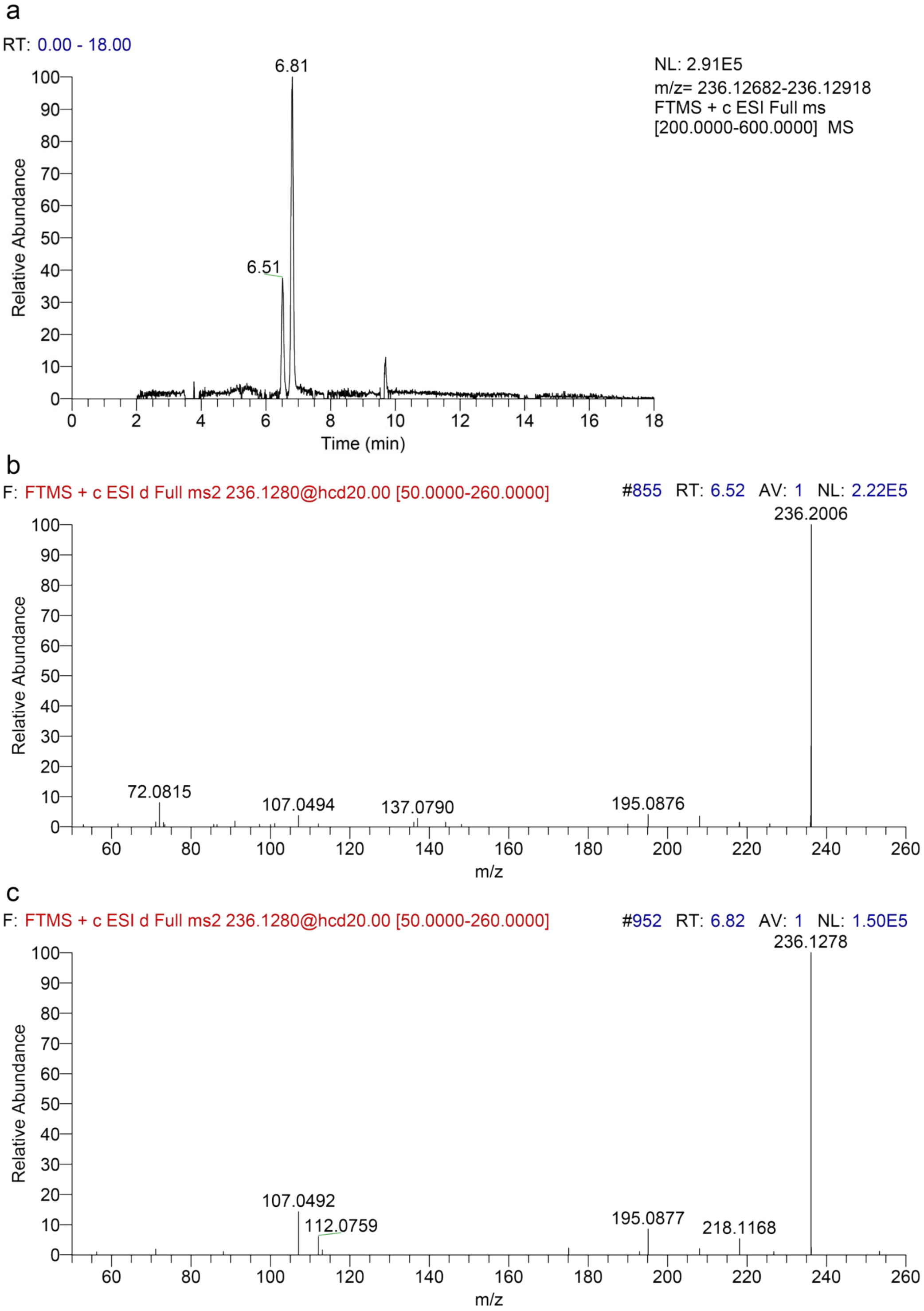

Figure S2. Extracted ion chromatogram of two isomeric forms of PMFTH$_2$.

a Extracted ion chromatogram corresponding to PMFTH$_2$ (*m/z* 236.12812 $[M+H]^+$), showing two isomeric forms eluting at 6.51 (minor form) and 6.81 min (major form). **b** MS/MS spectrum of the minor isomer eluting at 6.52 min. **c** MS/MS spectrum of the dominant isomer eluting at ca 6.82 min. Near identical mass and MS/MS fragmentation and similar retention time suggest that the two isomers represent tautomers of each other.

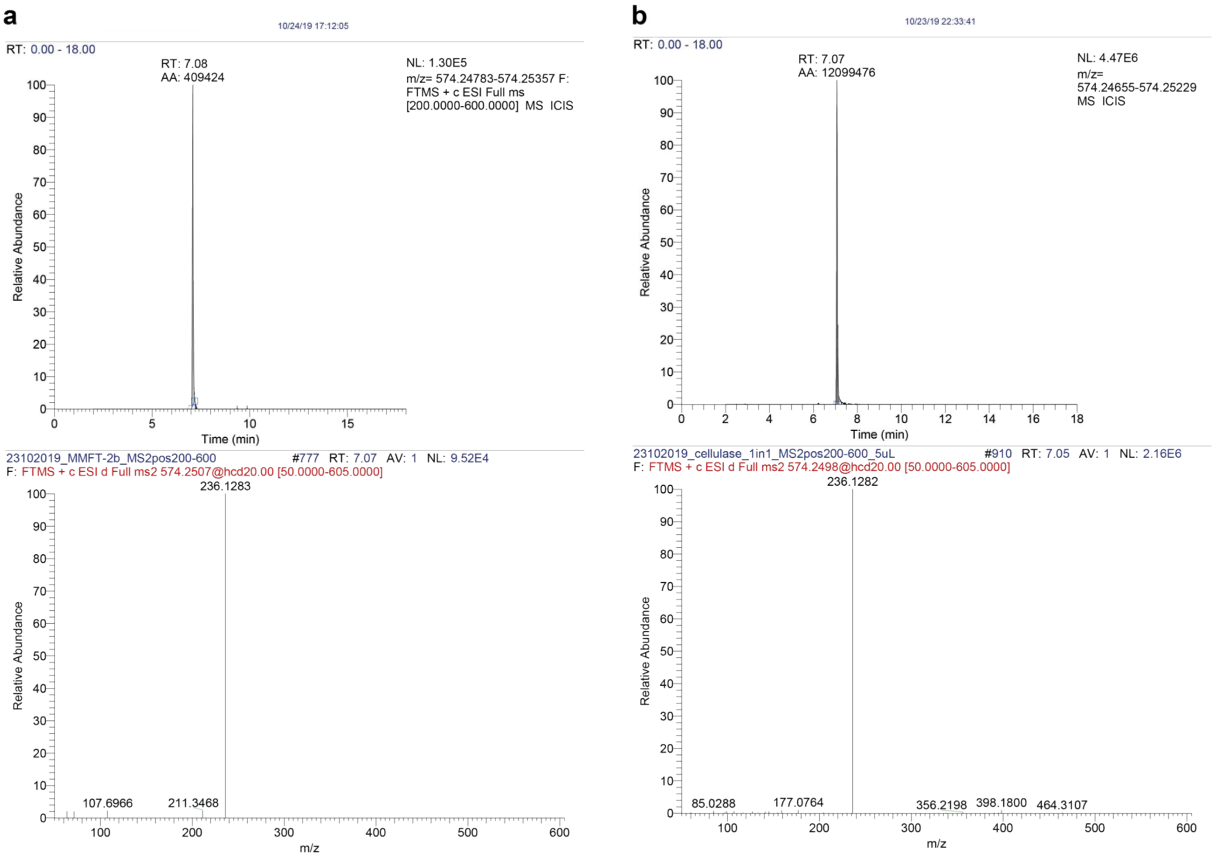

Figure S3. Extracted ion chromatogram and mass spectrum of MMFT-2bH$_2$.

a MMFT-2bH$_2$ enriched from a large-scale cultivation (50 L) performed in a fermentor. The extracted ion chromatogram shows MMFT-2bH2 ([M+H]$^+$ *m/z* 574.24640). The corresponding MS/MS spectrum is shown below. **b** MMFT-2bH$_2$ generated by cellulase treatment of mycofactocin extracts. The extracted ion chromatogram shows MMFT-2bH2 ([M+H]$^+$ *m/z* 574.24640). The corresponding MS/MS spectrum is shown below. Both compounds are identical according to LC-MS/MS.

GC-MS analyses of the oligosaccharide moiety

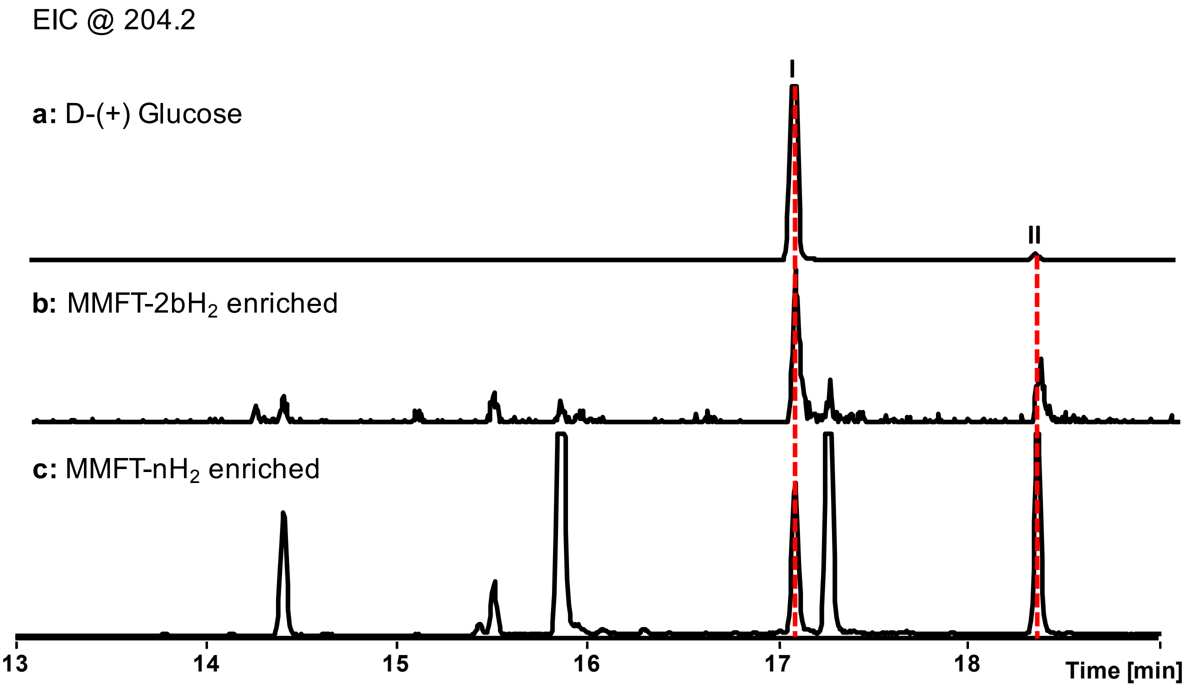

Figure S4. Proposed monosaccharide composition in MMFT.
a GC-MS chromatograms of D-(+)-glucose after MSTFA derivatization. **b** acid hydrolysis of MMFT-2bH$_2$ enriched fractions by 3 M HCl (aq.) and their perTMS derivatives. **c** acid hydrolysis of MMFT-n enriched fractions by 3 M HCl (aq.) and their perTMS derivatives. Compound I (t_R = 17.3 min) and II (t_R = 18.4 min): glucose-1,2,3,4,6-OTMS (GC-MS chromatograms were displayed under EIC mode at *m/z* 204.2, which represented one of the diagnostic fragment ions of hexose-1,2,3,4,6-OTMS. MSTFA: *N*-methyl-*N*-(trimethylsilyl)trifluoroacetamide. TMS: trimethylsilyl. It should be noted that due to the low abundance of MMFT it was only possible to use enriched fractions for GC-MS analysis so that polysaccharide contaminations remained visible in the analysis.

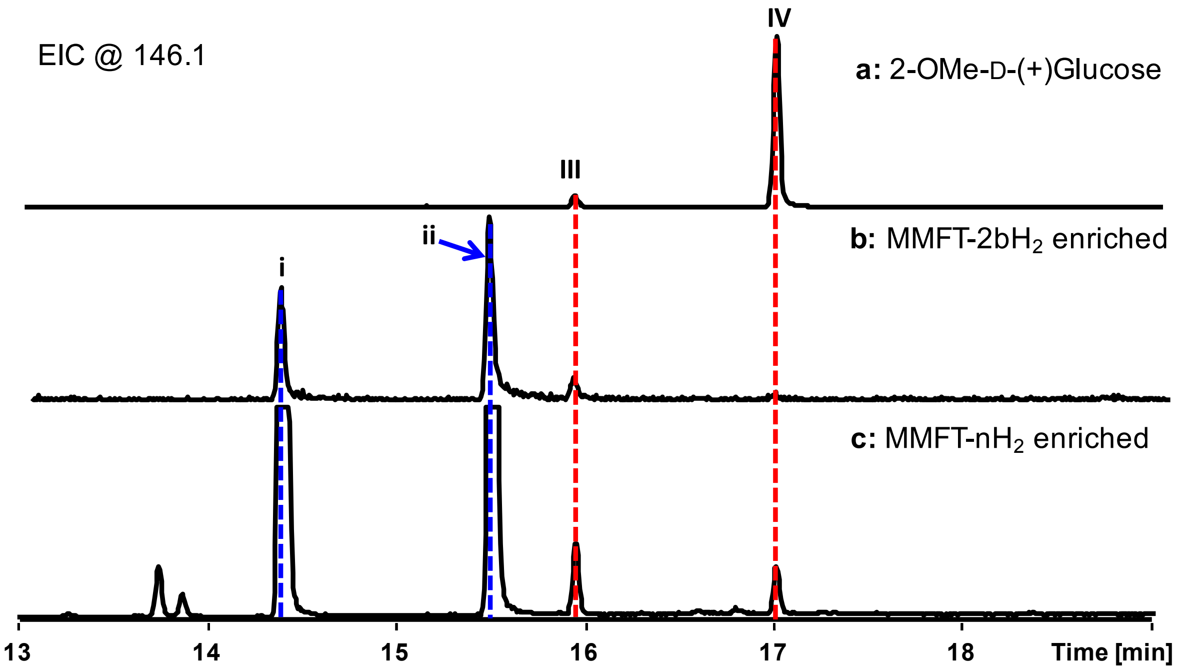

Figure S5. Proposed methylated monosaccharide composition in MMFT. a GC-MS chromatograms of 2-OMe-D-(+)-glucose after MSTFA derivatization. **b** acid hydrolysis of MMFT-2bH$_2$ enriched fractions by 3 M HCl (aq.) and their perTMS derivatives. **c** acid hydrolysis of MMFT-n enriched fractions by 3 M HCl (aq.) and their perTMS derivatives. **Compound III** (t_R = 16.0 min) and **IV** (t_R = 17.0 min): glucose-2-OMe-1,3,4,6-OTMS (GC-MS chromatograms were displayed under EIC mode at *m*/*z* 146.1, which represented one of the diagnostic fragment ions of hexose-2-OMe-1,3,4,6-OTMS. **Compound i** (t_R = 14.4 min) and **ii** (t_R = 15.5 min): hexose-3-OMe-1,2,4,6-OTMS derived from polysaccharide contaminations.

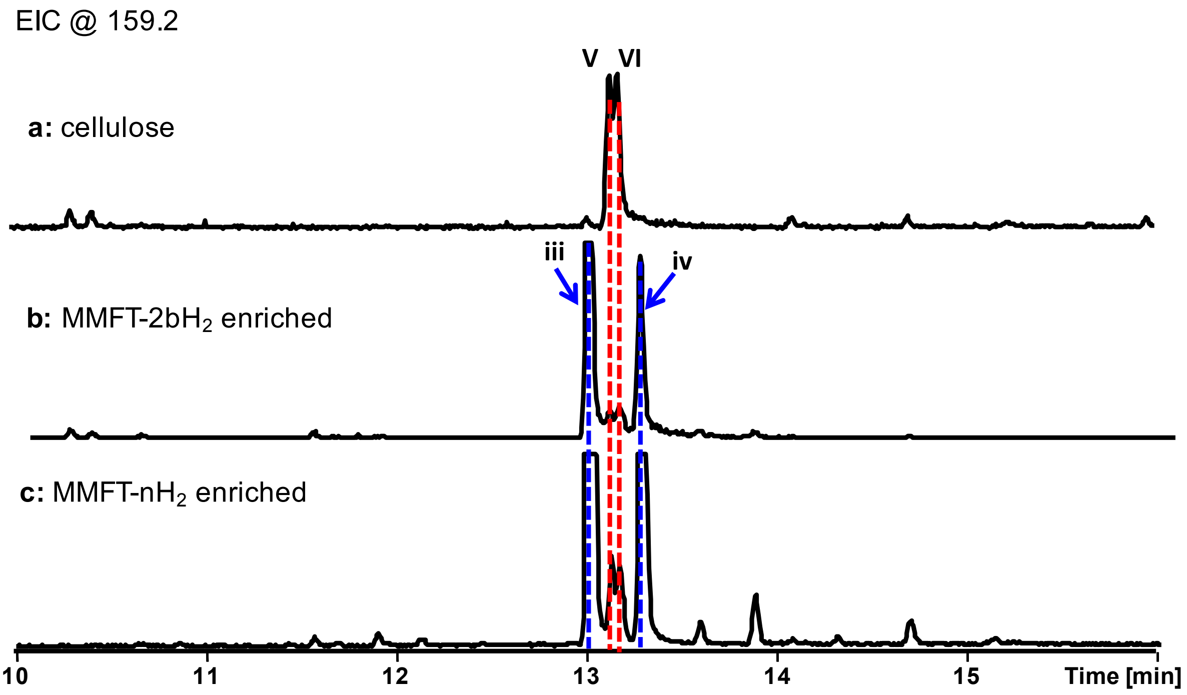

Figure S6. Proposed monosaccharide linkage in MMFT.

a GC-MS chromatogram of cellulose after permethylation and acid hydrolysis by 3 M HCl (aq.) and MSTFA derivatization. **b** MMFT-2bH$_2$ enriched fraction after permethylation and acid hydrolysis by 3 M HCl (aq.) and MSTFA derivatization. **c** MMFT-n enriched fraction after permethylation and acid hydrolysis by 3 M HCl (aq.) and MSTFA derivatization. **Compound V** (t_R = 13.21 min) and **VI** (t_R = 13.26 min) : glucose-2,3,6-OMe-1,4-OTMS (GC-MS chromatograms were displayed under EIC mode at *m*/*z* 159.2, which represented one of the diagnostic fragment ions of hexose-2,3,6-OMe-1, 4-OTMS. **Compound iii** (t_R = 13.0 min) and **iv** (t_R = 13.3 min): hexose-2,3,6-OMe-1,4-OTMS derived from polysaccharide contaminations.

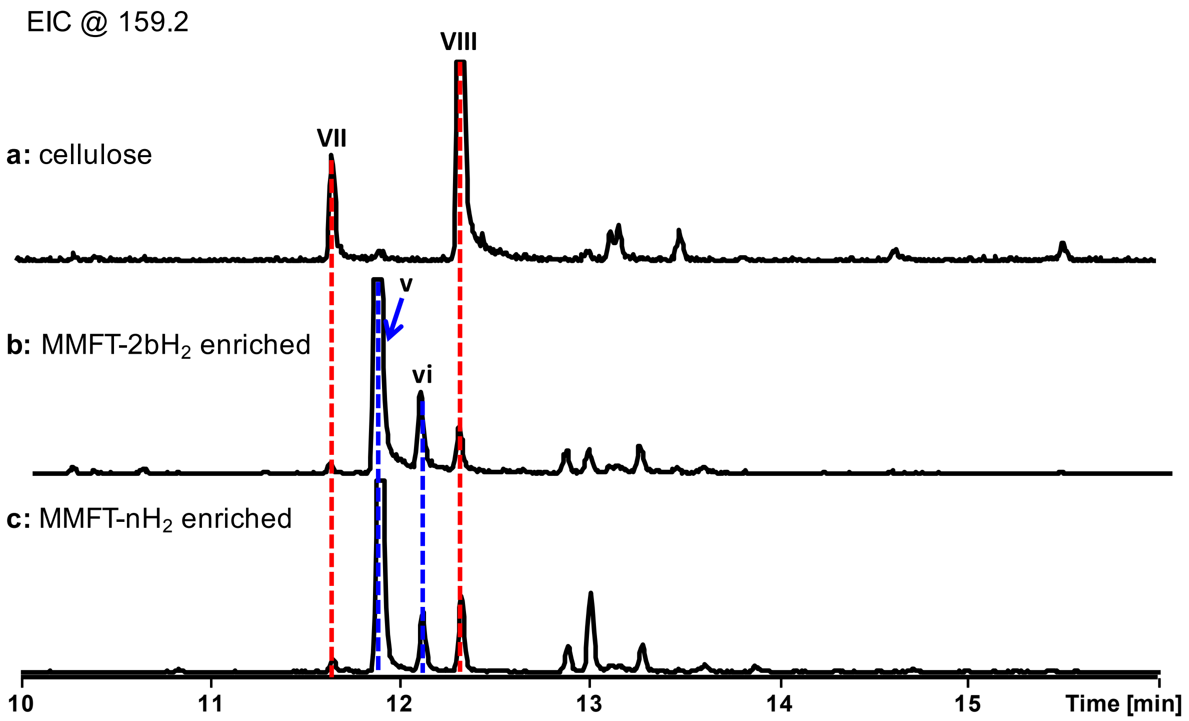

Figure S7. Proposed monosaccharide linkage in MMFT.

a GC-MS chromatogram of cellulose after permethylation and methanolysis by 1.25 M HCl (in MeOH) and MSTFA derivatization. **b** MMFT-2bH$_2$ enriched fraction after permethylation and methanolysis by 1.25 M HCl (in MeOH) and MSTFA derivatization **c** MMFT-n enriched fraction after permethylation and methanolysis by 1.25 M HCl (in MeOH) and MSTFA derivatization. **Compound VII** (t_R = 11.7 min) and **VIII** (t_R = 12.3 min): glucose-1,2,3,6-OMe-4-OTMS (GC-MS chromatograms were displayed under EIC mode at *m*/*z* 159.2, which represented one of the diagnostic fragment ions of hexose-1,2,3,6-OMe-4-OTMS. **Compound v** (t_R = 11.9 min) and **vi** (t_R = 12.1 min): hexose-1,2,3,6-OMe-4-OTMS derived from polysaccharide contaminations.

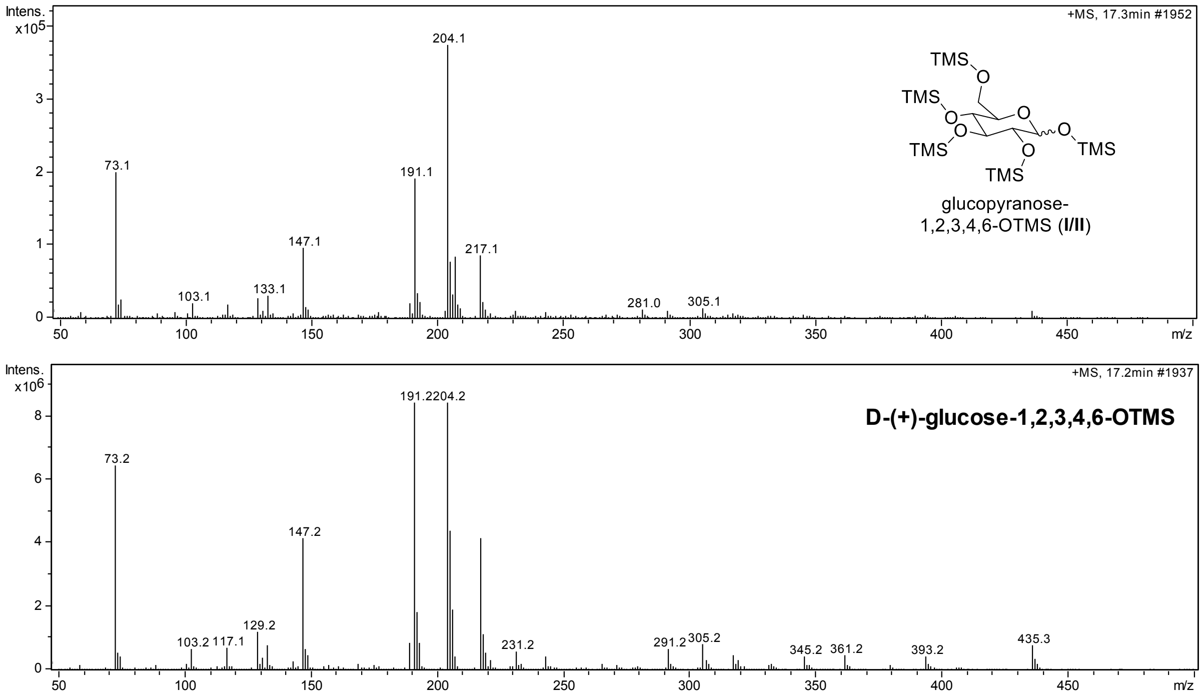

Figure S8. EI-MS spectra of compound I and standard D-(+)-glucose-1,2,3,4,6-OTMS.

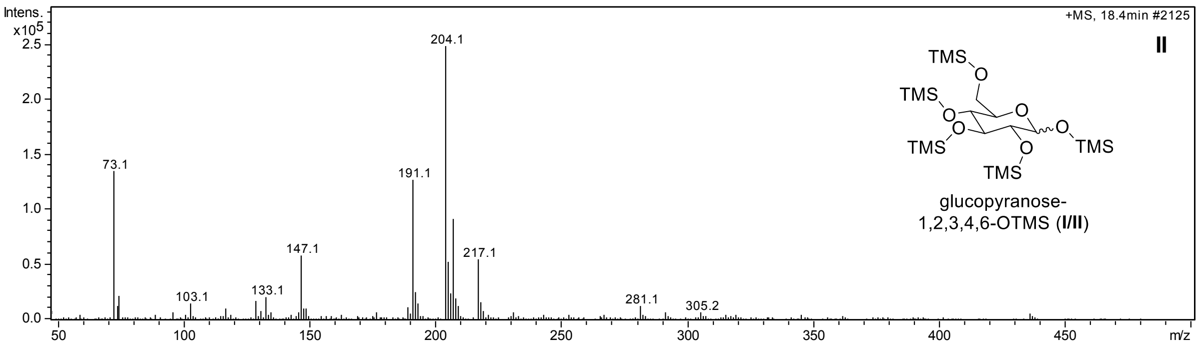

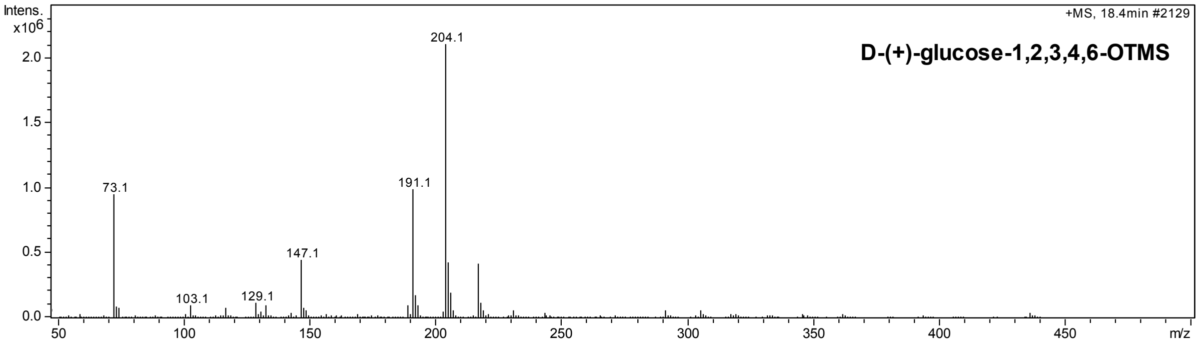

Figure S9. EI-MS spectra of compound II and standard D-(+)-glucose-1,2,3,4,6-OTMS.

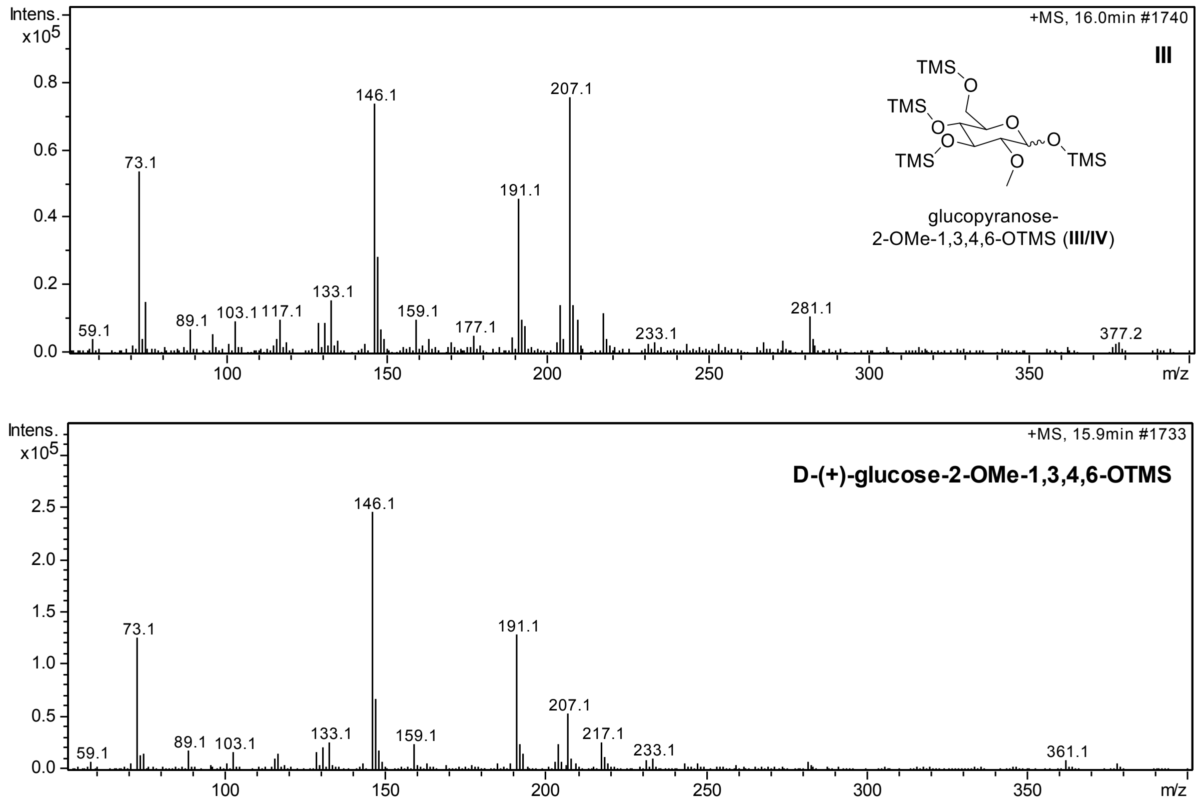

Figure S10. EI-MS spectra of compound III and standard D-(+)-glucose-2-OMe-1,3,4,6-OTMS

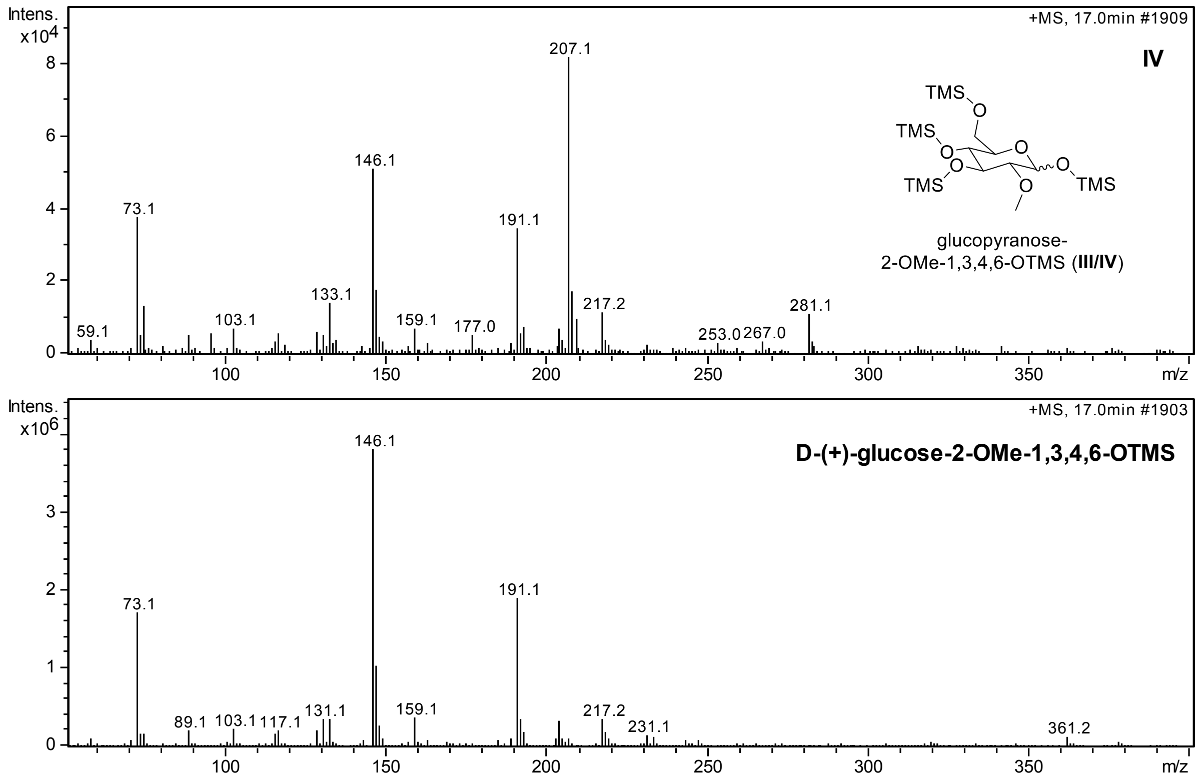

Figure S11. EI-MS spectra of compound IV and standard D-(+)-glucose-2-OMe-1,3,4,6-OTMS

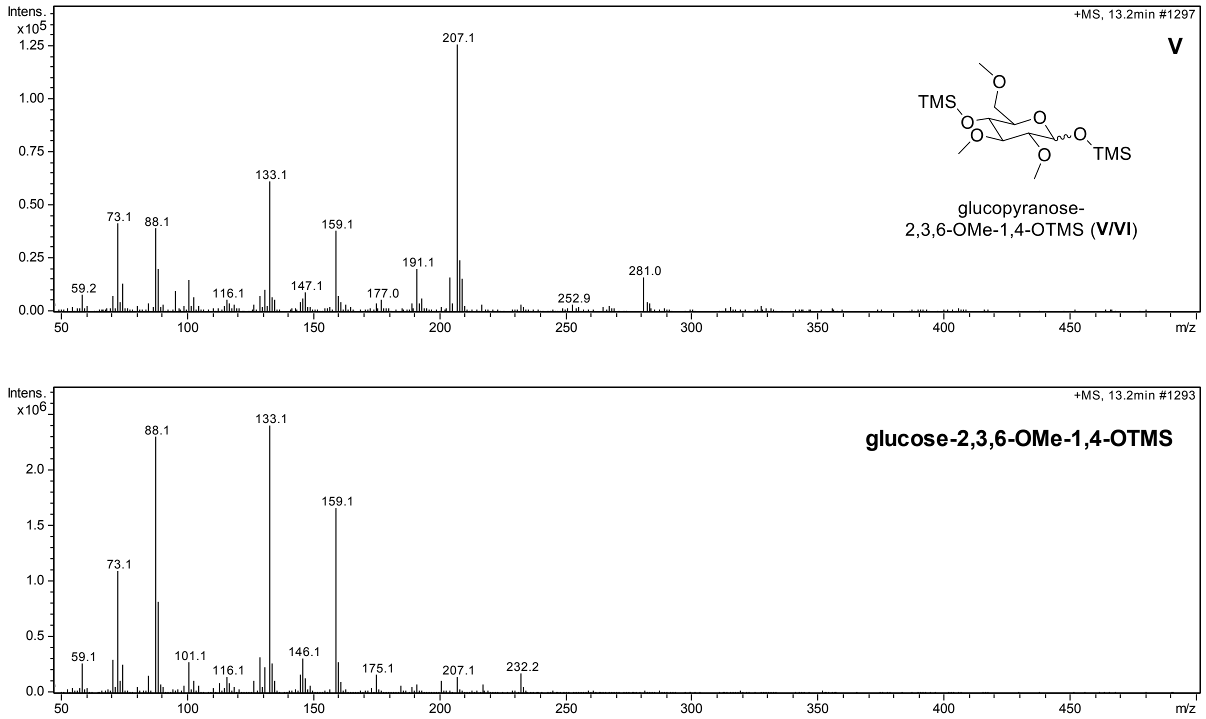

Figure S12. EI-MS spectra of compound V and glucose-2,3,6-OMe-1,4-OTMS derived from cellulose

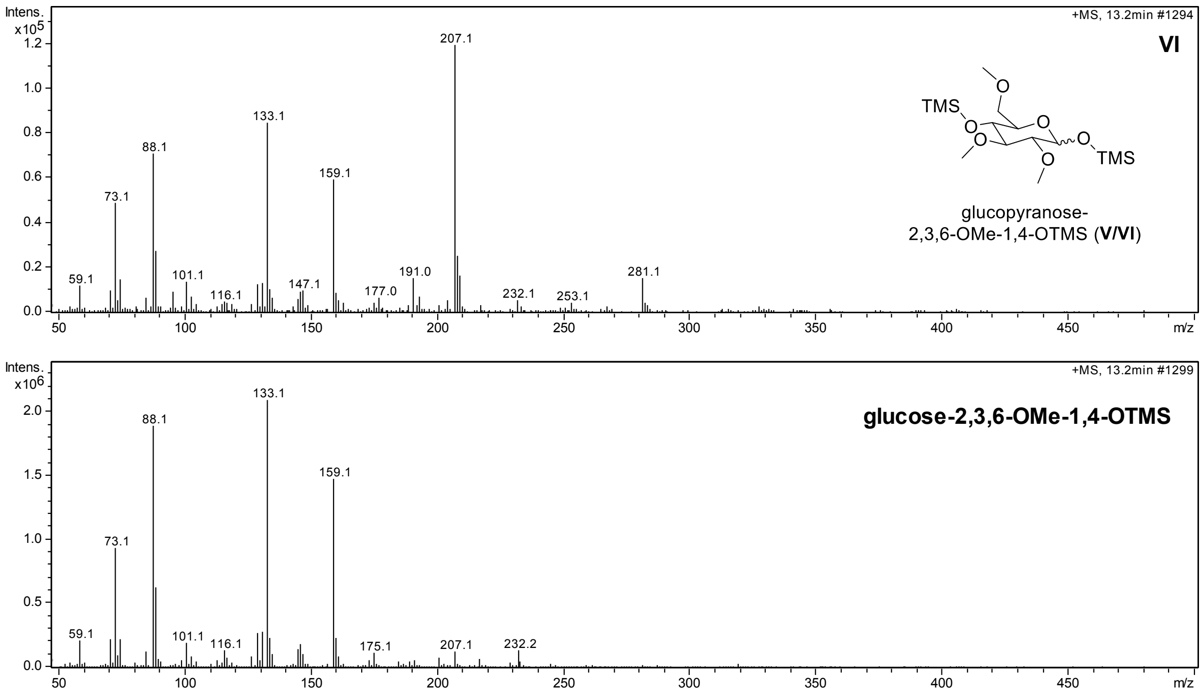

Figure S13. EI-MS spectra of compound VI and glucose-2,3,6-OMe-1,4-OTMS derived from cellulose

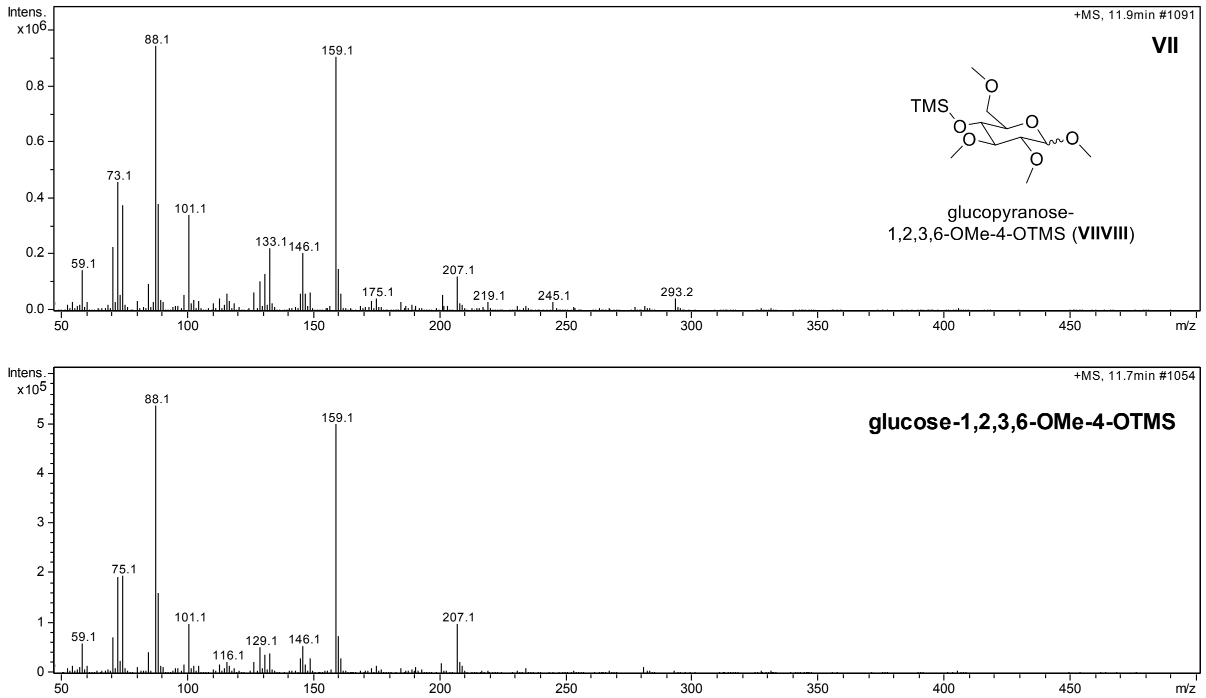

Figure S14. EI-MS spectra of compound VII and glucose-1,2,3,6-OMe-4-OTMS derived from cellulose

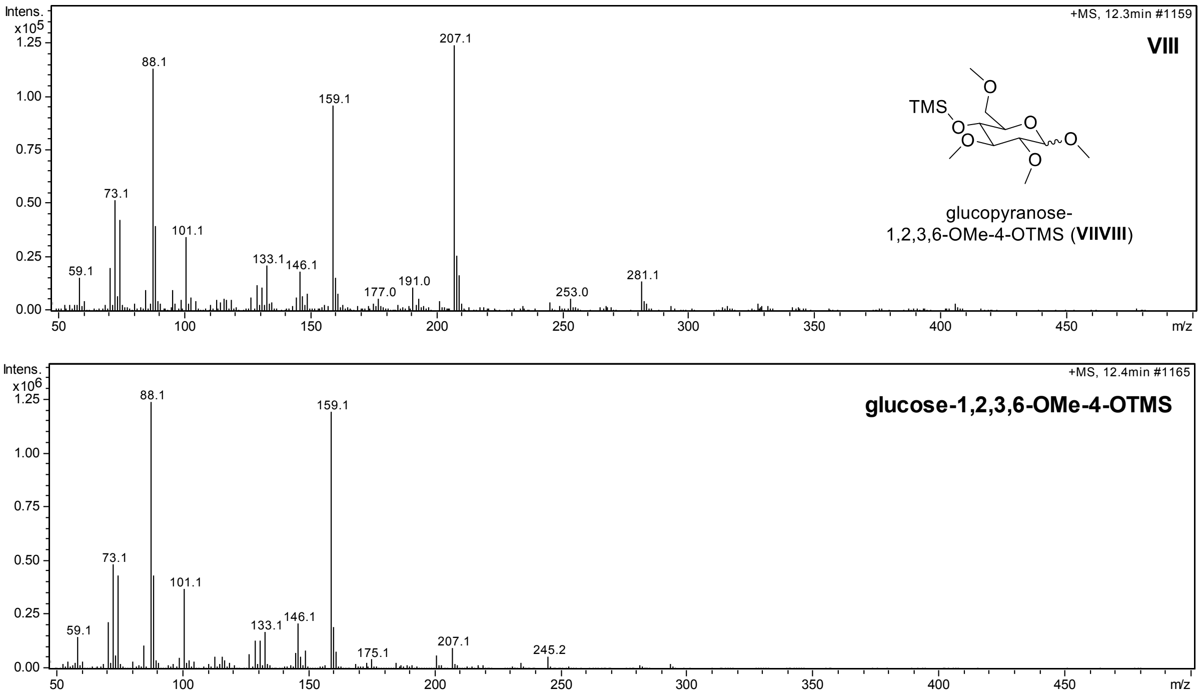

Figure S15. EI-MS spectra of compound VIII and glucose-1,2,3,6-OMe-4-OTMS derived from cellulose

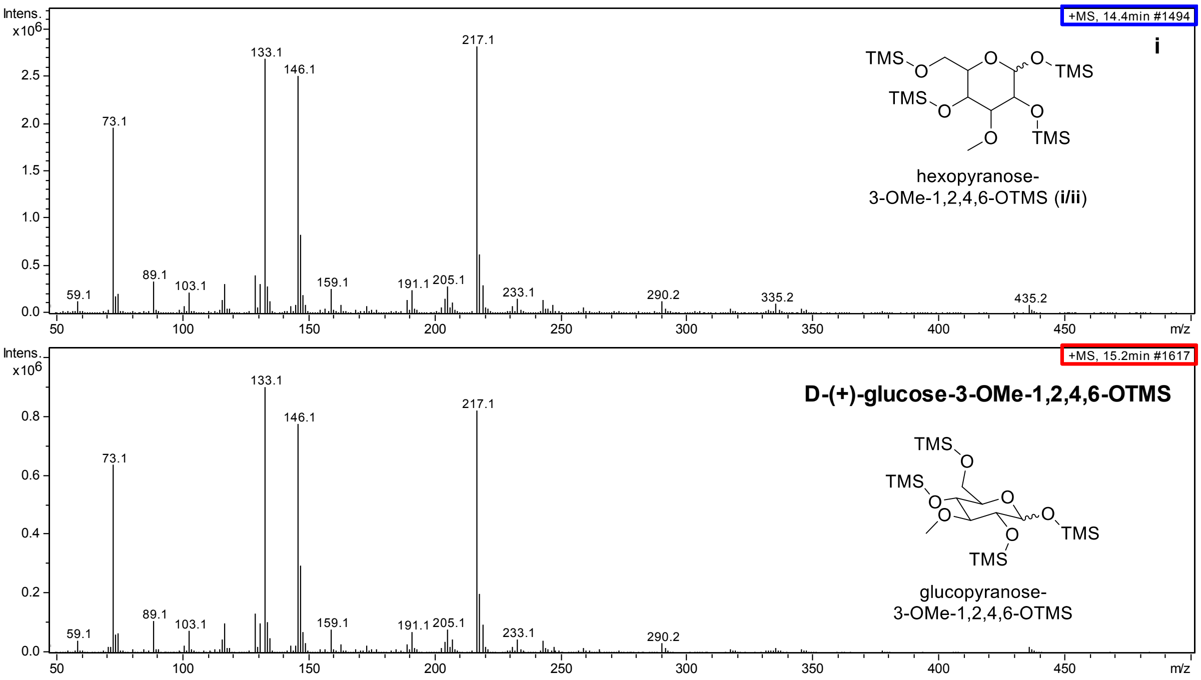

Figure S16. EI-MS spectra of compound i and standard D-(+)-glucose-3-OMe-1,2,4,6-OTMS

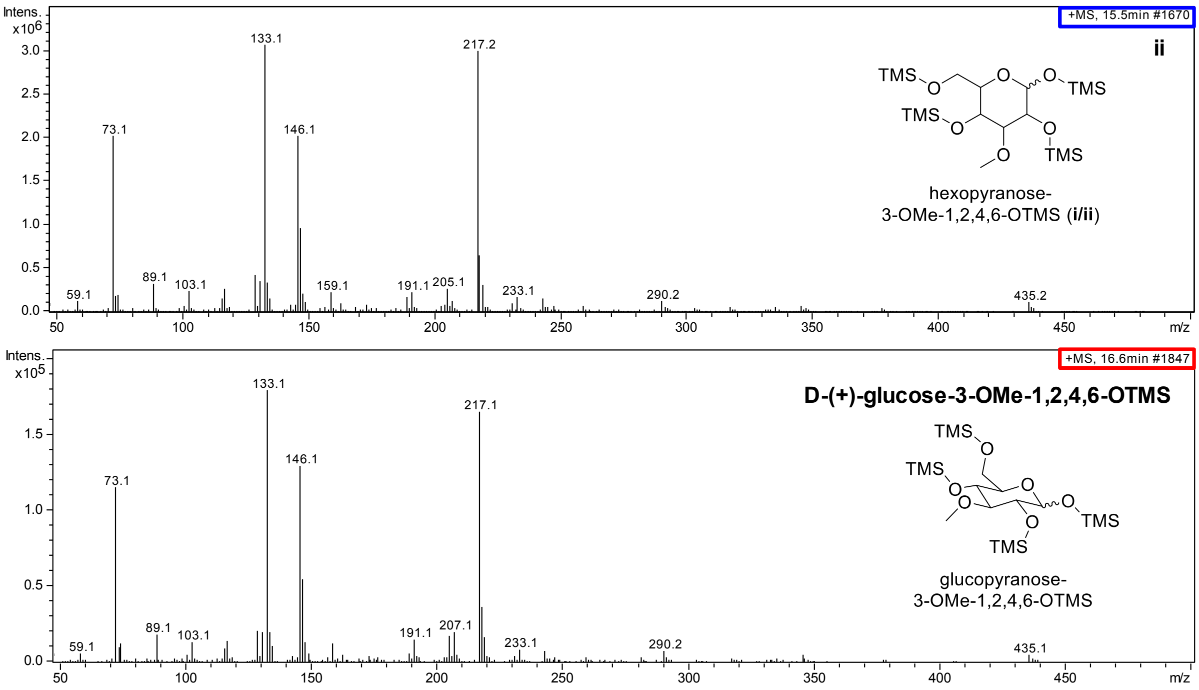

Figure S17. EI-MS spectra of compound ii and standard D-(+)-glucose-3-OMe-1,2,4,6-OTMS

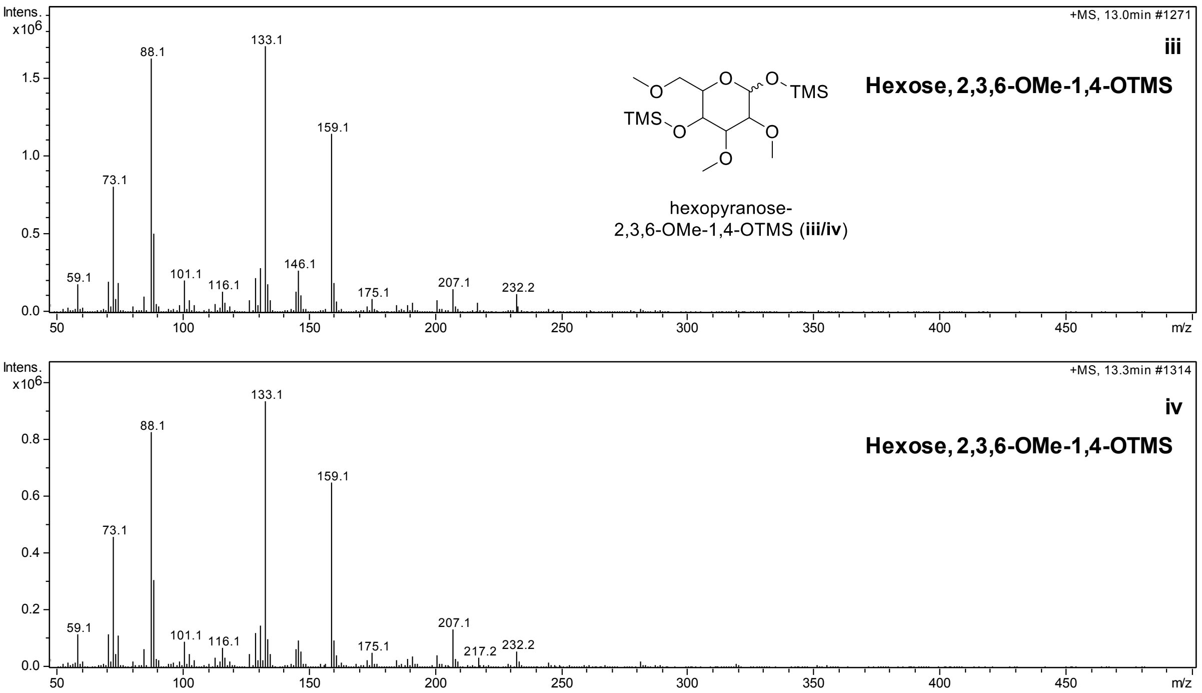

Figure S18. EIMS spectra of compounds iii and iv

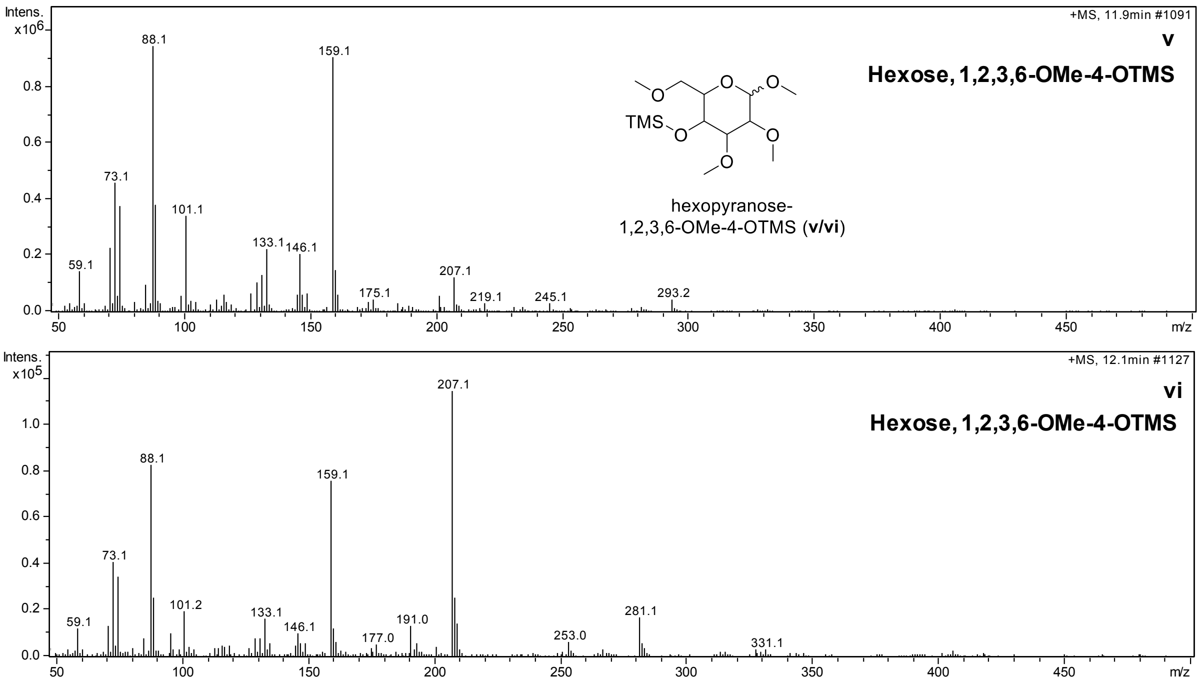

Figure S19. EI-MS spectra of compounds v and vi

Structure elucidation of MMFT-7/8-H_2 by NMR

Purified MMFT-7/8-H_2 mixture was firstly suspended into D_2O: ND_4OD (v/v 10:1; ND_4OD is 25 wt. % in D_2O) to improve solubility. The standard 1D NMR (^1H NMR and ^{13}C NMR) and 2D NMR spectra (COSY, HSQC, HMBC and NOESY) were recorded on a Bruker 600 MHz spectrometer equipped with cryo probe (Figures S20 – S27, Table S1 and S2). By detailed comparison with the reported NMR data of AHDP and PMFT(Ayikpoe & Latham, 2019), the five-membered lactam moiety was deduced by the observation of two non-equivalent methyl groups (δ_{H-6} 1.06 ppm/δ_{C-6} 21.44 ppm; δ_{H-7} 1.25 ppm/δ_{C-7} 20.30 ppm), an isolated methine group connected with hydroxyl group (δ_{H-3} 4.28 ppm/δ_{C-3} 76.16 ppm) and proton within a substituted lactam (δ_{H-1} 3.50 ppm/δ_{C-1} 63.03 ppm). The assignment of the lactam moiety was completed based on HMBC correlations of H-3 to C-4 (δ_C 178.09 ppm) and C-2 (δ_C 42.43 ppm) and C-6, H-1 to C-2 and C-6. Furthermore, the HMBC correlation of H_3-7 to C-1, C-2, C-3, C-6 and H_3-6 to C-1, C-2, C-3, C-7 confirmed the five-membered lactam moiety, biosynthetically derived from cyclization of valine and tyrosine. The complete tyrosine moiety was deduced by the observation of one methylene moiety (δ_{H2-5} 3.01ppm, 2.61 ppm/δ_{C-5} 36.21 ppm) and two sets of *para*-substituted aromatic protons (δ_{H-9} 7.25 ppm/δ_{C-9} 130.63 ppm; δ_{H-10} 7.12 ppm/δ_{C-9} 116.71 ppm). The tyrosine moiety was assigned based on the COSY correlations of H-1 to H_2-5 as well as H-9 to H-10, and further confirmed by the HMBC correlations of H_2-5 to C-1 and C-9, H-9 to C-5 and C-11 (δ_C 155.40 ppm), H-10 to C-8 (δ_C 133.90 ppm) and C-11. So far, the core planar structure of mycofactocin in its reduced form was assigned unambiguously.

The attached sugar moieties were deduced by the observation of overlapping oxymethine and oxymethylene moieties (δ_H 3.3 – 4.0 ppm/δ_C 59.87 – 82.50 ppm). The first anomeric carbon C-1' ($\delta_{H-1'}$ 5.16 ppm/$\delta_{C-1'}$ 100.12 ppm) was assigned based on the HMBC correlation of H-1' to C-11. The second anomeric carbon C-1'' ($\delta_{H-1''}$ 4.63 ppm/$\delta_{C-1'}$ 102.35 ppm) was assigned by the COSY correlation of H-1'' to H-2'' ($\delta_{H-2''}$ 3.15 ppm/$\delta_{C-2''}$ 82.50 ppm), and HMBC correlation of H-1'' to C-2'', whose attached hydroxyl group supposed to be methylated ($\delta_{H-7''}$ 3.64 ppm/$\delta_{C-7''}$ 60.55 ppm). This hypothesis was confirmed by the HMBC correlation of H-7'' to C-2''. The anomeric carbon C-1''' ($\delta_{H-1'''}$ 4.56 ppm/$\delta_{C-1'''}$ 102.35 ppm) representing the rest of repeated glucose units was assigned by the obviously high integration value compared with H-1' and H-1''. The COSY correlations of H-1''' to H-2''', H-2''' to H-3''', H-5''' to H-6''', and the HMBC correlations of H-2''' to C-1'''/C-3''', H-3''' to C-4''', H-4''' to C-3'''/C-5''', H-5''' to C-3'''/C-4''' and H-6''' to C-4''' allowed to assigned the glucose units. The glycosidic linkage was deduced by the HMBC correlation of H-1'' to C-4' and H-1''' to C-4''. The configuration of the glucose moiety was deduced as ß-form by the large coupling constant of anomeric protons ($J_{H-1'-H-2'}$ = 8.0 Hz; $J_{H-1''-H-2''}$ = 8.0 Hz; $J_{H-1'''-H-2'''}$ = 8.0 Hz). With above observations, the attached sugar moieties were assigned as ß-1,4 glucose, with the second glucose is 2-oxymethyl-glucose. The planar structure and key HMBC correlations were presented in Figure S27.

The trace amount of oxidized form of MMFT-7/8 was recognized by the observation of *para*-substituted aromatic protons at δ_H 7.34 ppm (d, 8.31 Hz)/δ_C 130.62 ppm, δ_H 7.14 ppm (d, 8.00 Hz)/δ_C 116.74 ppm, one anomeric carbon (δ_H 4.53 ppm/δ_C 102.40 ppm) from the glucose unit, one oxymethylene carbon δ_H 3.94 ppm, 3.75 ppm/δ_C 60.88 ppm from the glucose unit, and two non-equivalent methyl groups (δ_H 0.96 ppm; δ_H 1.13 ppm).

Table S1. NMR data (D_2O/ND_4OD 10:1, at 300 K) of MMFT-7/8-H_2[a]

	**MMFT-7/8-H_2**				
position	δ_C, mult.[b]	δ_H, mult. (J in Hz)	COSY	HMBC	NOESY
1	63.03, CH	3.50, dd (10.93, 3.74)	5b	2, 6	5a, 7
2	42.43, qC				
3	76.16, CH	4.28, s		2, 4, 6	6
4	178.09, qC				
5	36.21, CH_2	3.01, dd (12.57, 3.29)		9	1, 5b, 7, 9
		2.61, t (12.57)	1	1, 9	5a, 7, 9
6	21.44, CH_3	1.06, s		1, 2, 3, 7	1, 3
7	20.30, CH_3	1.25, s		1, 2, 3, 6	5a, 5b
8	133.90, qC				
9	130.63, CH	7.25, d (8.48)	10	5, 11	
10	116.71, CH	7.12, d (8.48)	9	8, 11	
11	155.40, qC				
1‘	100.12, CH	5.16, d (8.00)	2‘	11	10, 6‘a
2‘	72.66, CH	3.63, t,	1‘‘		
3‘[d]	73.97, CH	3.68, m			
4‘[d]	78.27, CH	3.70, m			
5‘	74.78, CH	3.65, m			
6‘	59.87, CH_2	4.00, d (11.25)			10
		3.84, dd (12.48, 5.46)			
1‘‘	102.35, CH	4.63, d (8.00)	2‘‘	4‘	4‘
2‘‘	82.50, CH	3.15, t (8.38)	1‘‘, 3‘‘	3‘‘	3‘‘
3‘‘[d]	73.97, CH	3,71, m	2‘‘	2‘‘	
4‘‘[d]	78,27, CH	3.70, m		1‘‘‘	
5‘‘	74.78, CH	3.65, m			
6‘‘	59.87, CH_2	4.00, d (11.25)			
		3.84, dd (12.48, 5.46)			
7‘‘	60.55, CH_3	3.64, s		2‘‘	
1‘‘‘[c]	102.35, CH	4.56, d (8.00)	2‘‘‘	4‘‘	2‘‘‘, 3‘‘‘, 4‘‘, 6‘‘‘a/b
2‘‘‘[c]	72.91, CH	3.37, t (8.34)	1‘‘‘, 3‘‘‘	1‘‘‘, 3‘‘‘	1‘‘‘, 3‘‘‘
3‘‘‘[c]	73.97, CH	3.68, m	2‘‘‘	4‘‘‘	
4‘‘‘[c]	78.27, CH	3.70, m		1‘‘, 3‘‘‘, 5‘‘‘, 6‘‘‘	
5‘‘‘[c]	74.78, CH	3.65, m	6‘‘‘a	3‘‘‘, 4‘‘‘	
6‘‘‘[c]	59.87, CH_2	4.00, d (11.25)	5‘‘‘, 6‘‘‘b	4‘‘‘	1‘‘‘, 6‘‘‘b
		3.84, dd (12.48, 5.46)	6‘‘‘a	4‘‘‘	

[a] 600 MHz for ^{1}H NMR and 150 MHz for ^{13}C NMR

[b] numbers of attached protons were determined by analysis of 2D spectra.

[c] NMR resonance are overlapped by sugar chains and only one unit was presented.
[d] overlapping signals with repeat unit

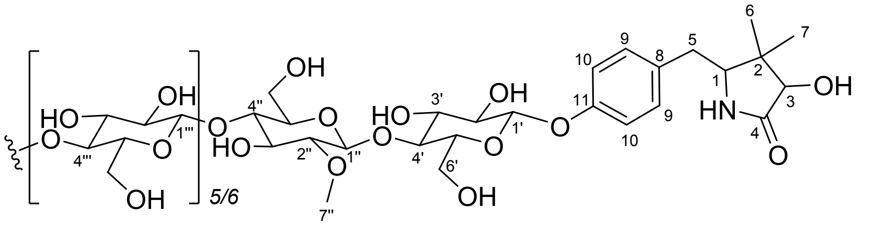

MMFT-7/8-H_2

Table S2. Comparative NMR data for peptide moiety of MMFT-7/8-H_2[a] and AHDP[b]

	MMFT-7/8-H_2		AHDP	
position	δ_C	δ_H	δ_C	δ_H
1	63.03	3.50, dd (10.93, 3.74)	62.45	3.56, dd (10.02, 4.33)
2	42.43		43.07	
3	76.16	4.28, s	61.64	3.30, s
4	178.09		177.33	
5	36.21	3.01, dd (12.57, 3.29)	33.29	2.79, dd (14.18, 4.36)
		2.61, t (12.57)		2.49, dd (14.17, 10.04)
6	21.44	1.06, s	22.47	0.82, s
7	20.30	1.25, s	14.10	1.03, s
8	133.90		130.33	
9	130.63	7.25, d (8.48)	130.20	7.14, d (8.52)
10	116.71	7.12, d (8.48)	115.51	6.79, d (8.54)
11	155.40		154.12	

[a] 600 MHz for ^{1}H NMR and 150 MHz for ^{13}C NMR, D_2O/ND_4OD (25% w/v in D_2O) 10:1
[a] 500 MHz for ^{1}H NMR and 125 MHz for ^{13}C NMR, D_2O (pL 7.5)

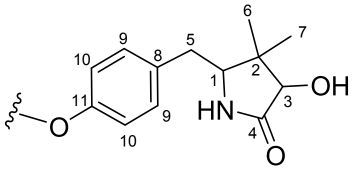

MMFT-H_2

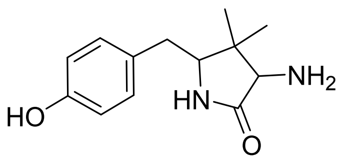

AHDP

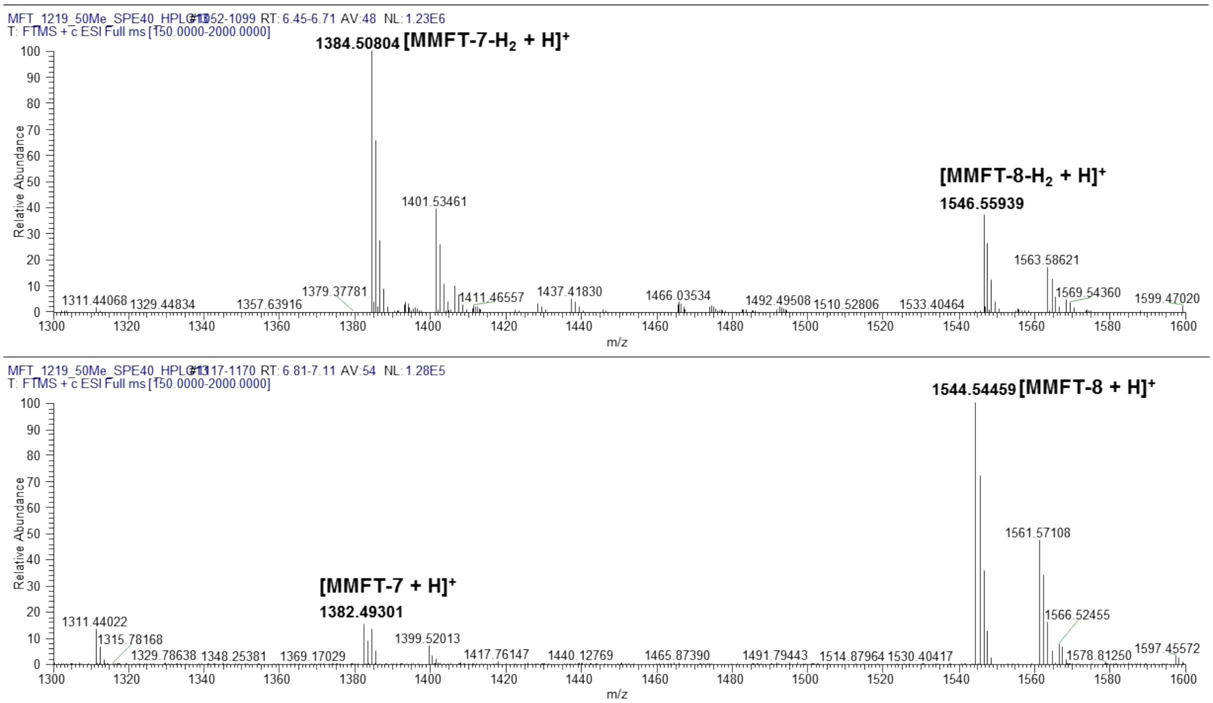

Figure S20. ESI-HRMS spectrum of MMFT-7/8-H_2 fraction

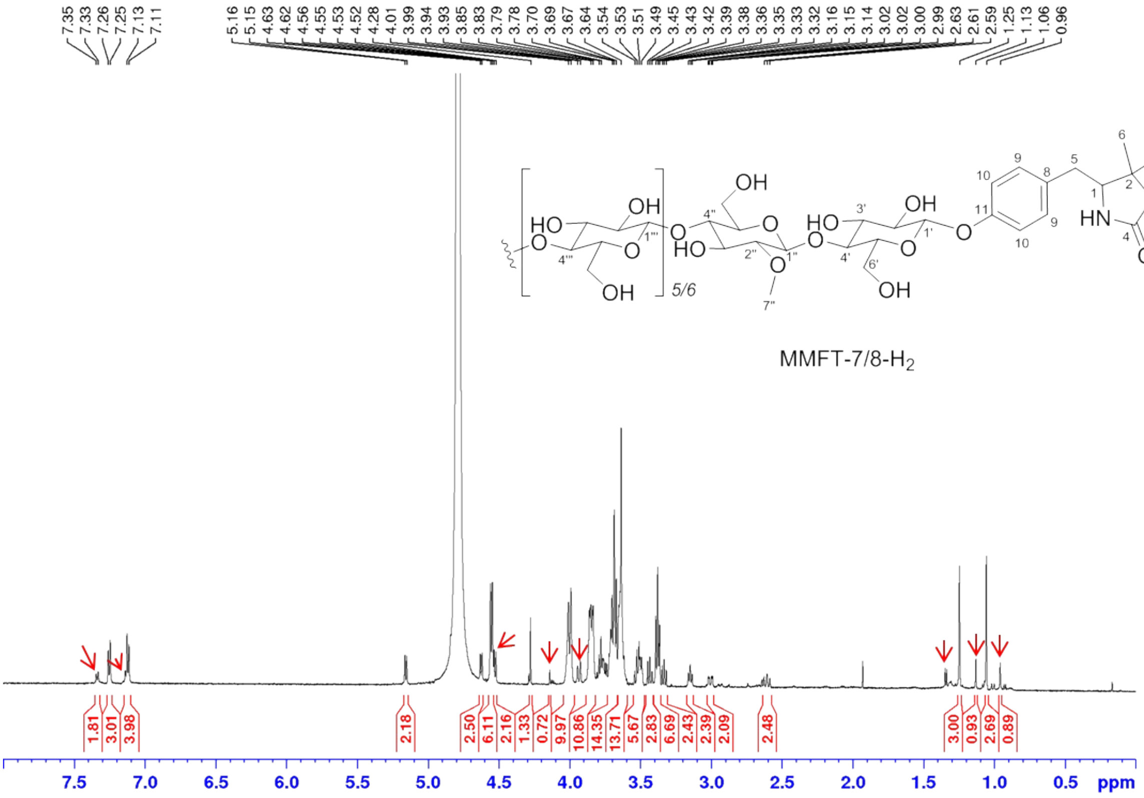

Figure S21. ^{1}H NMR spectrum of MMFT-7/8-H_2 (D_2O:ND_4OD (25% w/v in D_2O) 10:1 , 600 MHz, 300 K), (Red arrow indicates the proposed MMFT-7/8)

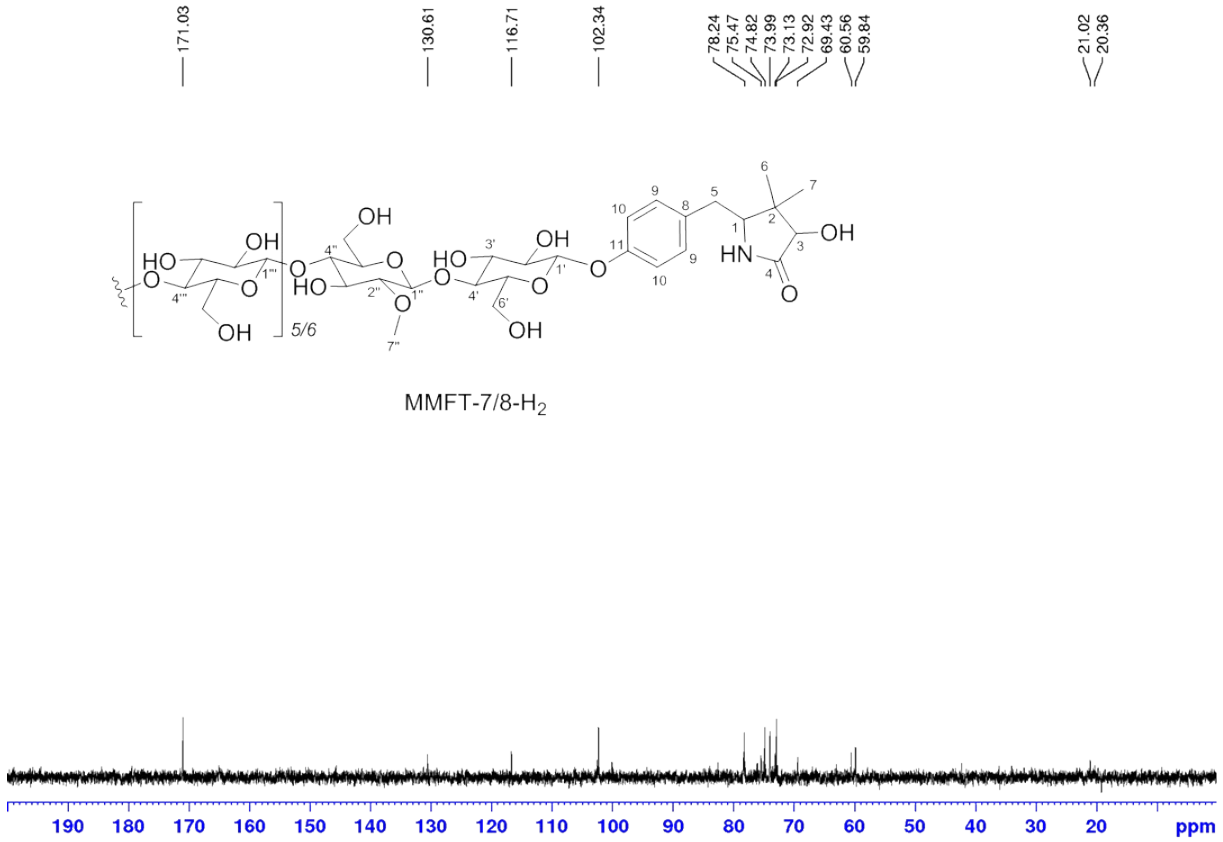

Figure S22. ^{13}C NMR spectrum of MMFT-7/8-H_2 (D_2O:ND_4OD (25% w/v in D_2O) 10:1 , 150 MHz, 300 K)

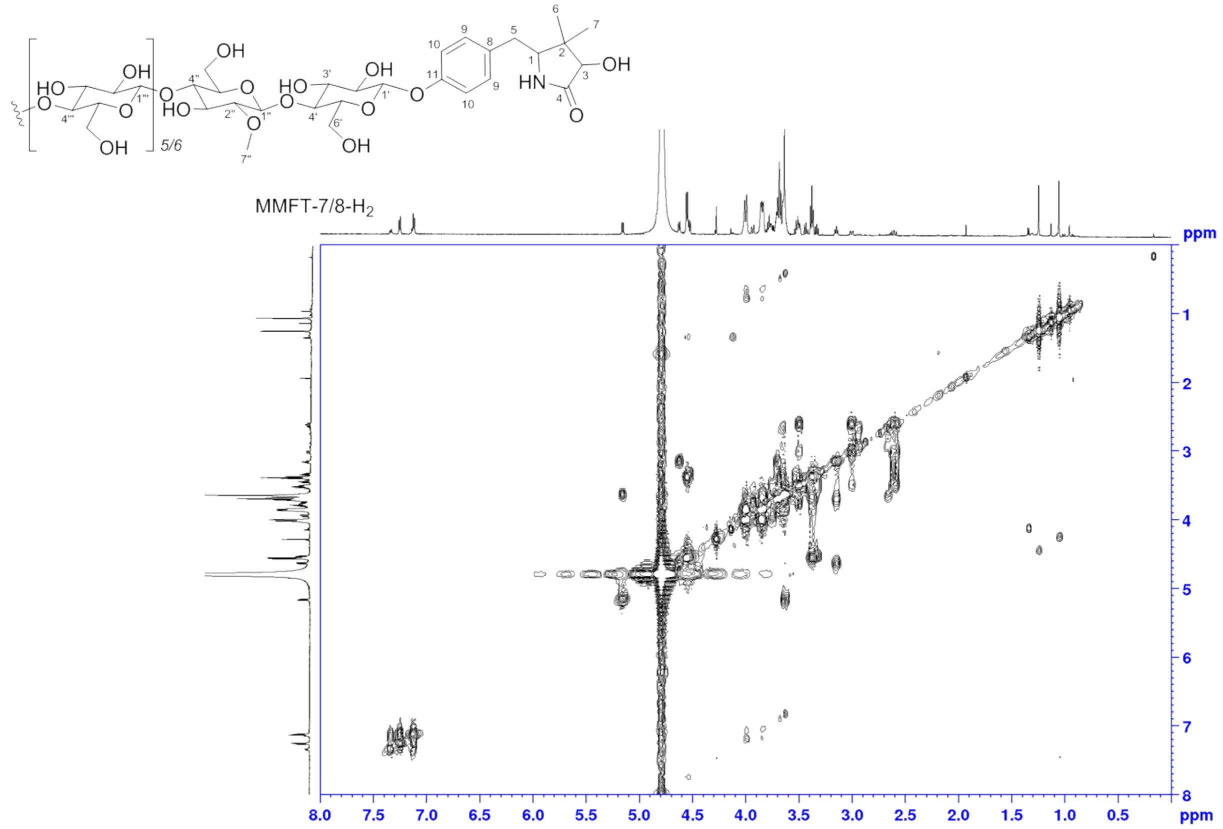

Figure S23. ^{1}H-^{1}H COSY spectrum of MMFT-7/8-H_2 (D_2O:ND_4OD (25% w/v in D_2O) 10:1 , 600 MHz, 300 K)

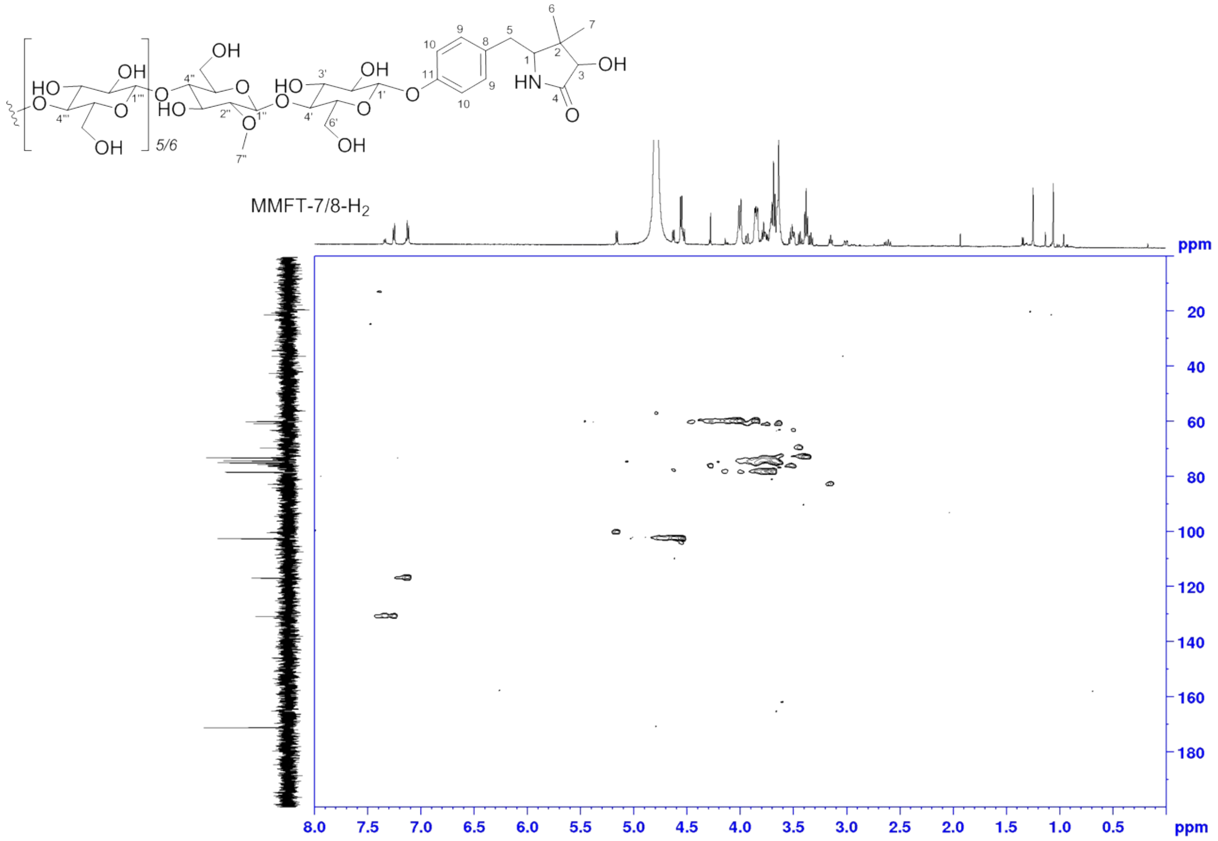

Figure S24. HSQC spectrum of MMFT-7/8-H_2 (D_2O:ND_4OD (25% w/v in D_2O) 10:1 , 600 MHz, 300 K)

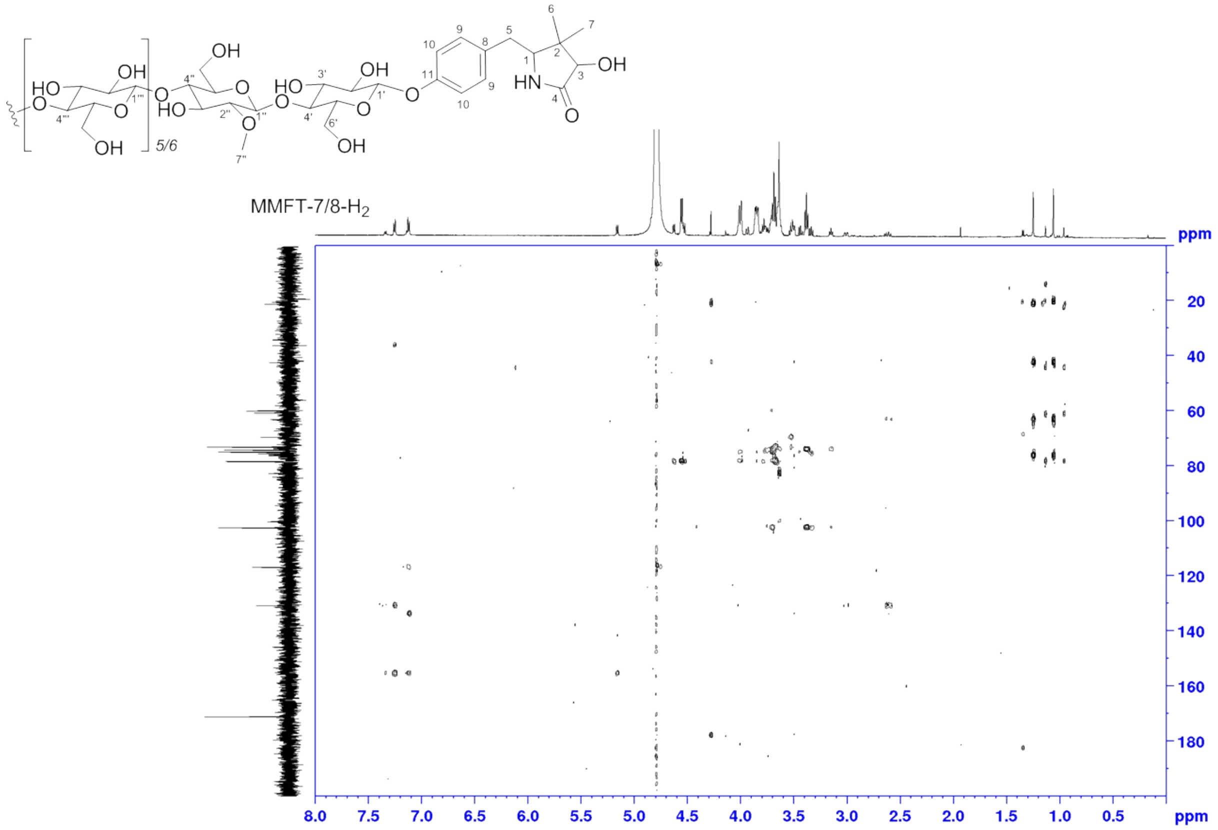

Figure S25. HMBC spectrum of MMFT-7/8-H_2 (D_2O:ND_4OD (25% w/v in D_2O) 10:1 , 600 MHz, 300 K)

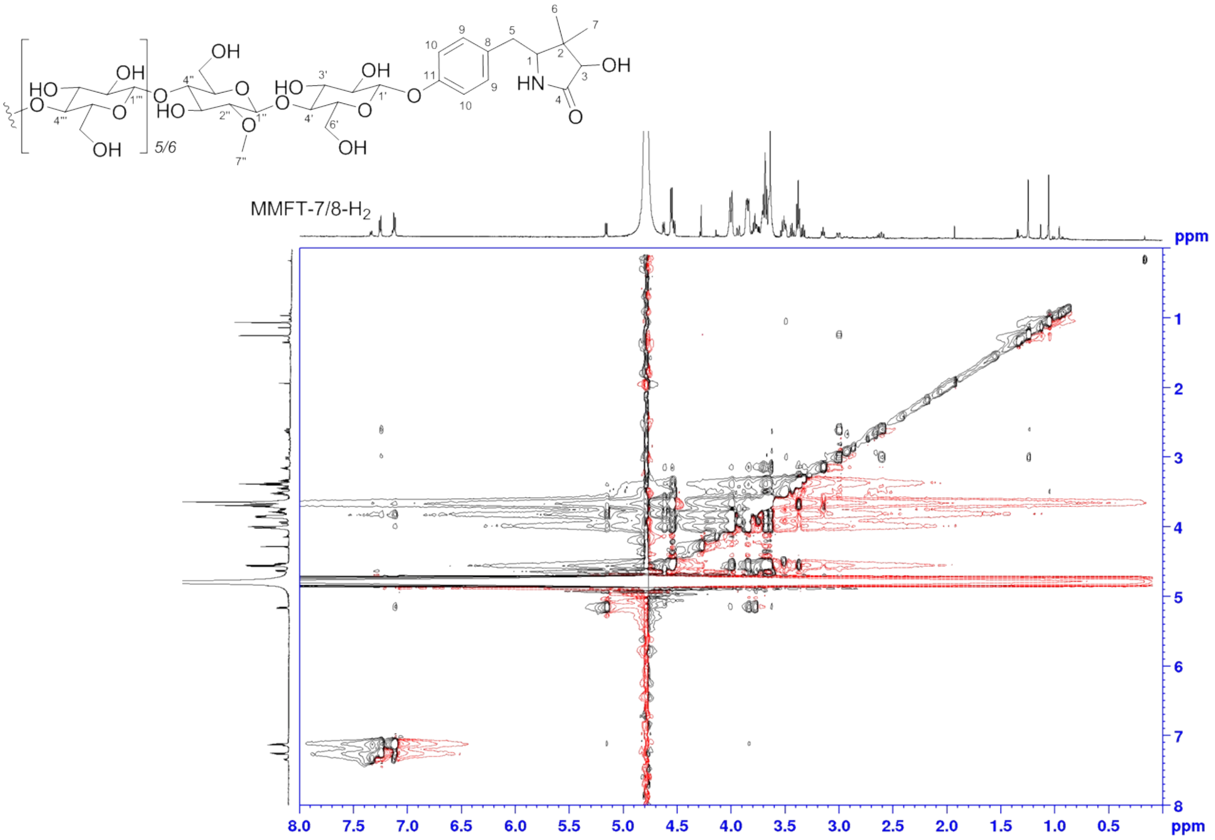

Figure S26. NOESY spectrum of MMFT-7/8-H$_2$ (D_2O:ND_4OD (25% w/v in D_2O) 10:1, 600 MHz, 300 K)

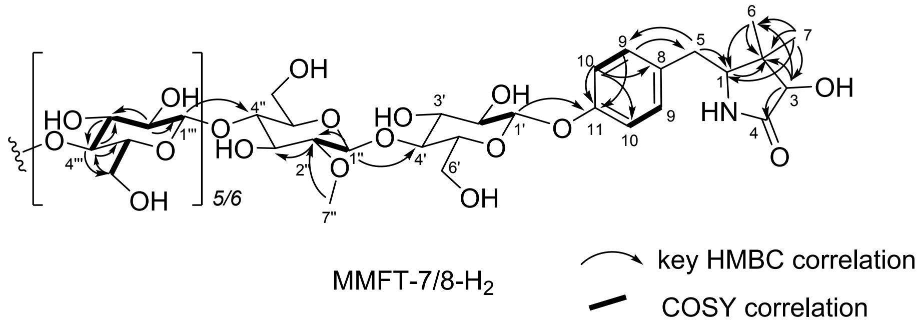

Figure S27. HMBC correlations and COSY correlations of MMFT-7/H$_2$.

Additional results

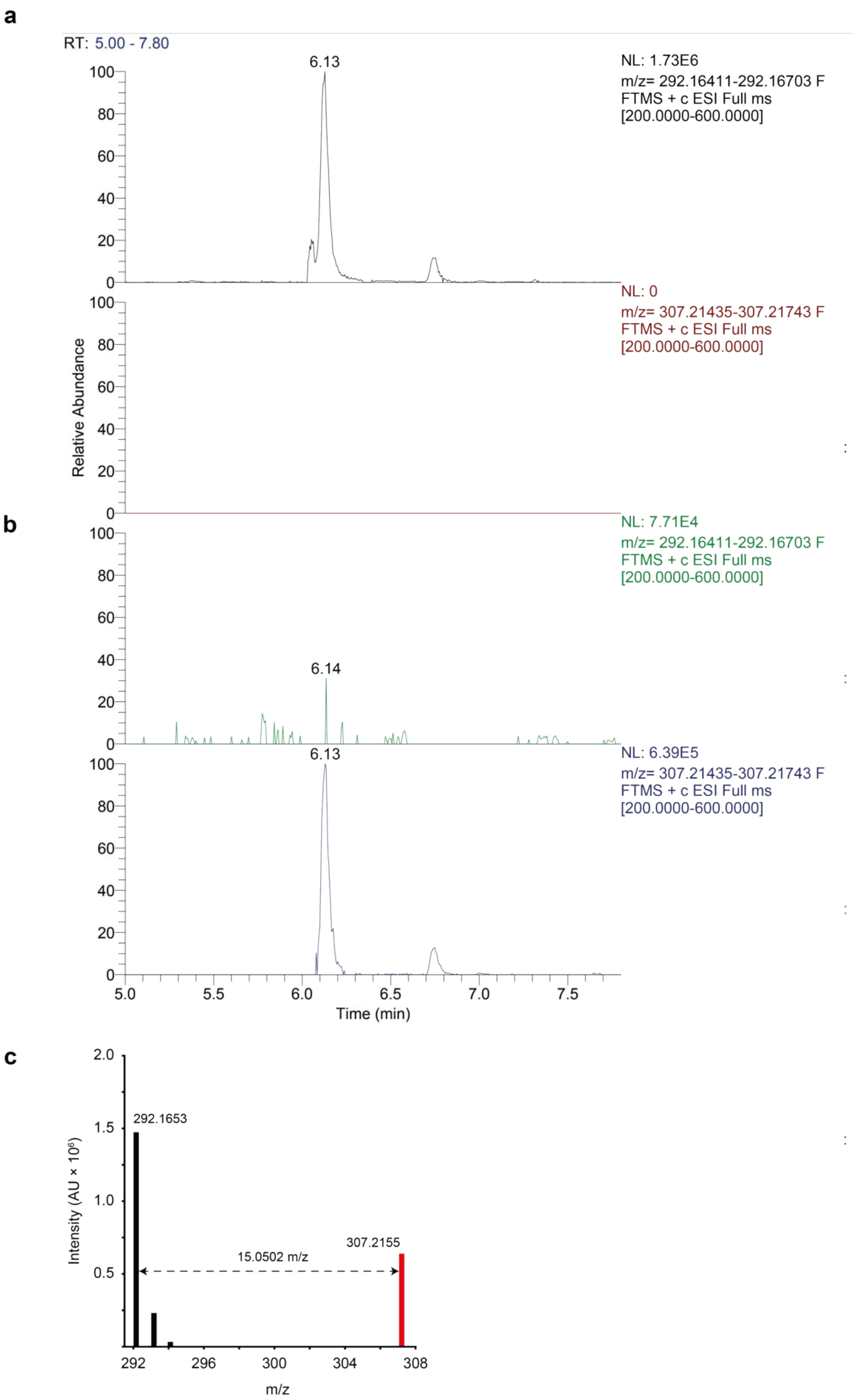

Figure S28. Stable isotope labeling of GAHDP.
Extracted ion chromatograms of unlabeled (*m/z* 292.16557 $[M+H]^+$) and labeled (*m/z* 307.21589 $[M+H]^+$) GAHDP from **a** *M. smegmatis* fed with unlabeled Gly, Val, and Tyr. **b** *M. smegmatis* fed with ^{13}C-labeled Gly-$^{13}C_2$, Val-$^{13}C_5$, and Tyr-$^{13}C_9$. **c** Overlaid mass spectra of unlabeled (black) and labeled GAHDP (red). Labeled GAHDP ($C_{15}H_{21}N_3O_3$) showed a mass shift of +15.05033 Da indicating the incorporation of all three labeled amino acids.

a

b

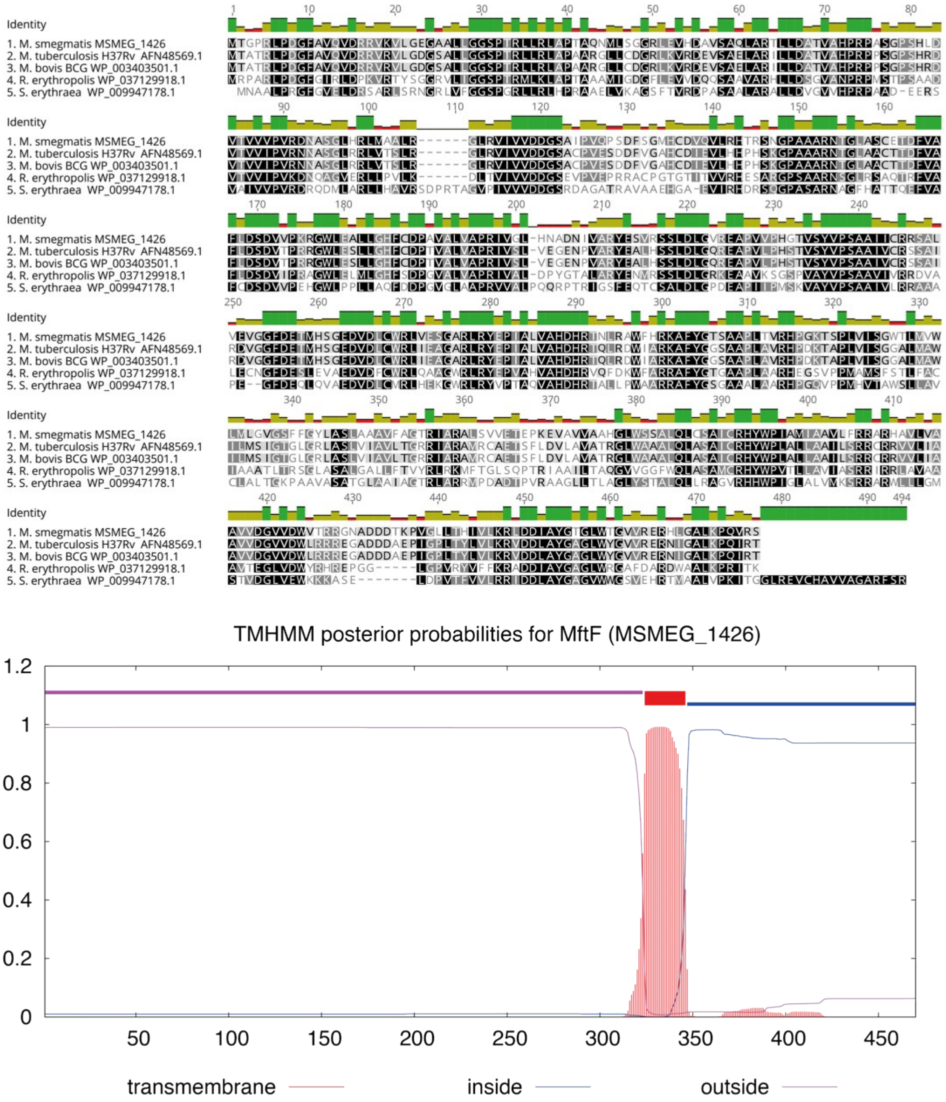

Figure S29. **Bioinformatics analyses of the MftF protein (MSMEG_1426).**

a Sequence alignment (MUSCLE algorithm) of MftF homologues. Source organisms: *Mycolicibacterium smegmatis* MC^2 155, *Mycobacterium tuberculosis* H37Rv, *Mycobacterium bovis* BCG BCG Pasteur 1173P2, *Rhodococcus erythropolis* SK121, *Saccharopolyspora erythraea* NRRL2338. Protein accession numbers are indicated together with sequence names. **b** Transmembrane helix prediction (TMHMM) of MSMEG_1426 revealed a membrane helix spanning residues 24 – 46.

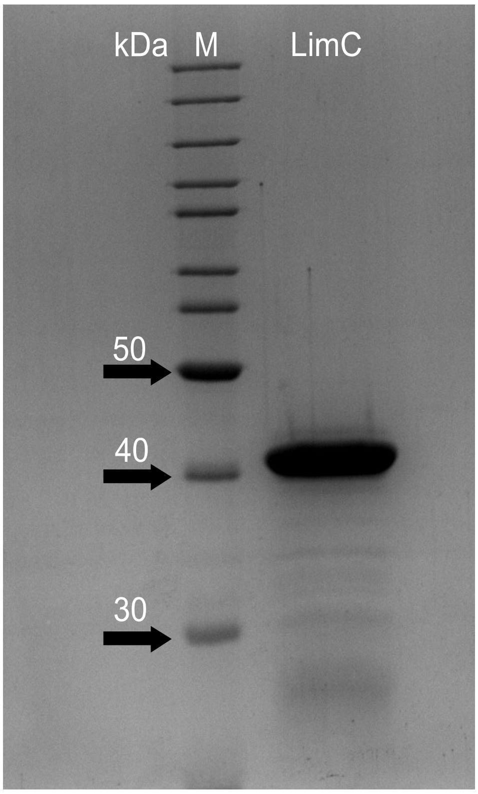

Figure S30. **SDS-PAGE of recombinant carveol dehydrogenase LimC from *Rhodococcus erythropolis* DCL14.** The hexahistidine-tagged protein (His6-LimC, 33 kDa) was produced in *E. coli* BL21 (DE3) / pLAPO04 (Figure S31) and purified by immobilized metal-affinity chromatography using a nickel-nitrilacetic acid resin. The protein elutes at around 40 kDa due to reduced gel mobility. M = molecular weight marker (PageRuler, Thermo Fisher).

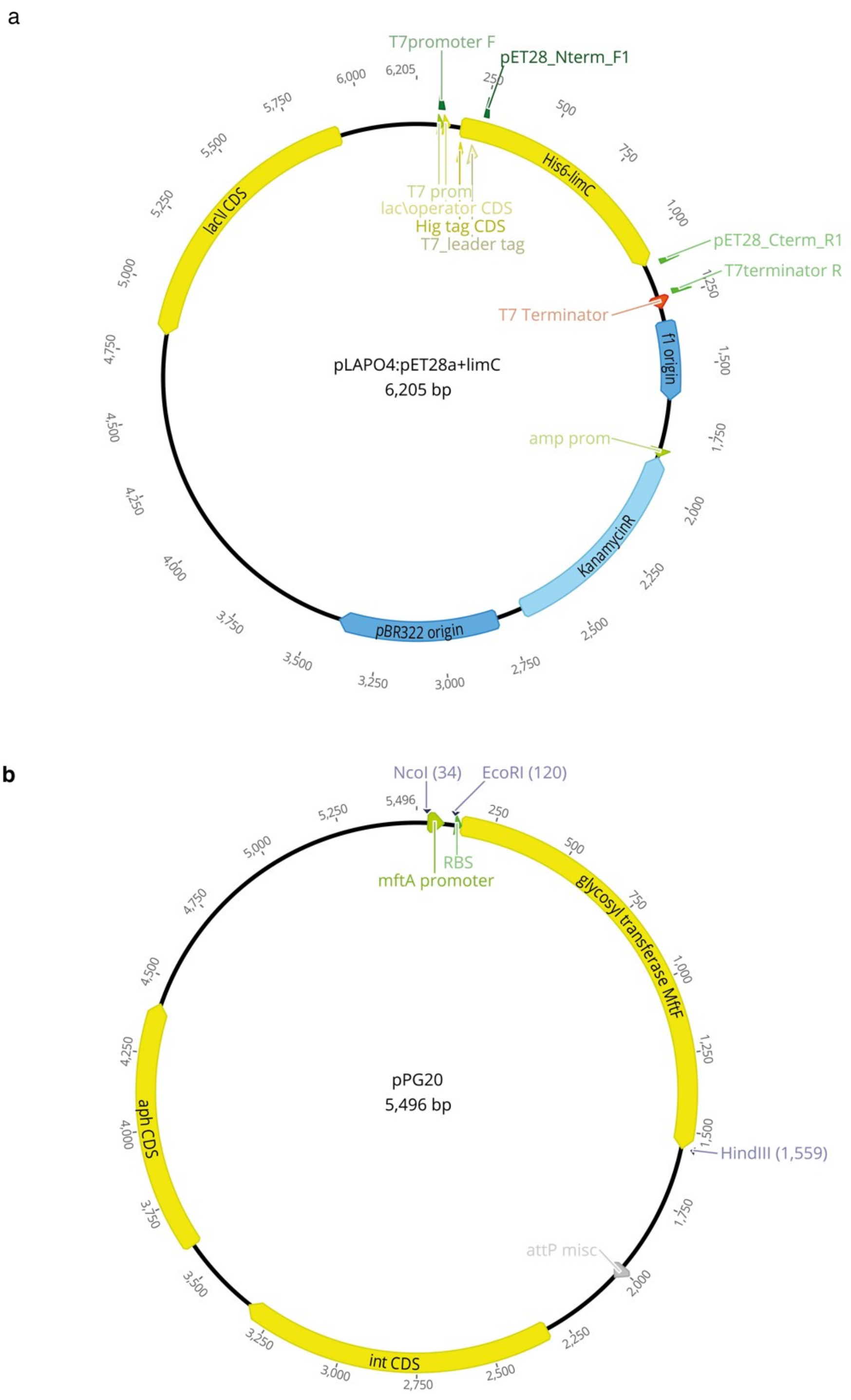

Figure S31. Maps of plasmids used in this study.
a Plasmid pLAPO04 used for heterologous expression of *limC* in *E. coli* BL21 (DE3): Vector pET28a bearing the coding sequence (CDS) of *limC* from *Rhodococcus erythropolis* DCL14 fused to the N-terminal His-tag region. **b** Plasmid pPG20 used for complementation of the *mftF* gene (Δ*mftF*-Comp) in *M. smegmatis*: Integrative vector pMCPAINT carries the promotor region and ribosome binding site (RBS) of *mftA* fused to the natural CDS of *mftF* for homologous expression in *M. smegmatis*. Promotor and RBS are separated by an additional *Eco*RI site. The whole insert was obtained as a synthetic construct flanked by *Nco*I and *Hind*III sites. Plasmid sequences are shown in Section 3.

Plasmid Sequences

>pLAPO4:pET28a+limC

GCGCCAGCAACCGCACCTGTGGCGCCGGTGATGCCGGCCACGATGCGTCCGGCGTAGAGGATCGAGATCTCGA
TCCCGCGAAATTAATACGACTCACTATAGGGGAATTGTGAGCGGATAACAATTCCCCTCTAGAAATAATTTTGTT
TAACTTTAAGAAGGAGATATACCATGGGCAGCAGCCATCATCATCATCATCACAGCAGCGGCCTGGTGCCGCGC
GGCAGCCATATGGCTAGCATGACTGGTGGACAGCAAATGGGTCGCGTCATGGCACGTGTTGAAGGTCAGGTTG
CACTGATTACCGGTGCAGCACGTGGTCAGGGTCGTAGCCATGCAATTAAACTGGCCGAAGAGGGTGCAGATGT
TATTCTGGTTGATGTTCCGAATGATGTGGTGGATATTGGTTATCCGCTGGGCACCGCAGATGAACTGGATCAGA
CCGCAAAAGATGTTGAAAATCTGGGTCGTAAAGCCATTGTTATTCATGCCGATGTTCGTGATCTGGAAAGCCTG
ACAGCCGAAGTTGATCGTGCAGTTAGCACCCTGGGTCGTCTGGATATTGTTAGCGCAAATGCAGGTATTGCAAG
CGTTCCGTTTCTGAGCCATGATATTCCGGATAATACCTGGCGTCAGATGATTGATATTAATCTGACCGGTGTTTG
GCATACCGCCAAAGTTGCAGTTCCGCATATTCTGGCAGGCGAACGTGGTGGTAGCATTGTTCTGACCAGCAGCG
CAGCAGGTCTGAAAGGTTATGCACAGATTAGCCATTATAGCGCAGCAAAACATGGTGTTGTTGGTCTGATGCGT
AGCCTGGCACTGGAACTGGCACCGCATCGTGTTCGTGTTAATAGCCTGCATCCGACACAGGTTAATACCCCGAT
GATTCAGAATGAAGGCACCTATCGTATTTTTAGTCCGGACCTGGAAAATCCGACACGTGAAGATTTTGAAATTGC
AAGCACCACCACCAATGCACTGCCGATTCCGTGGGTTGAAAGCGTTGATGTTAGCAATGCCCTGCTGTTTCTGGT
TAGCGAAGATGCACGTTATATTACAGGTGCCGCAATTCCGGTTGATGCAGGTACAACCCTGAAATAAGATCCGA
ATTCGAGCTCCGTCGACAAGCTTGCGGCCGCACTCGAGCACCACCACCACCACCACTGAGATCCGGCTGCTAAC
AAAGCCCGAAAGGAAGCTGAGTTGGCTGCTGCCACCGCTGAGCAATAACTAGCATAACCCCTTGGGGCCTCTAA
ACGGGTCTTGAGGGGTTTTTTGCTGAAAGGAGGAACTATATCCGGATTGGCGAATGGGACGCGCCCTGTAGCG
GCGCATTAAGCGCGGCGGGTGTGGTGGTTACGCGCAGCGTGACCGCTACACTTGCCAGCGCCCTAGCGCCCGC
TCCTTTCGCTTTCTTCCCTTCCTTTCTCGCCACGTTCGCCGGCTTTCCCCGTCAAGCTCTAAATCGGGGGCTCCCTT
TAGGGTTCCGATTTAGTGCTTTACGGCACCTCGACCCCAAAAAACTTGATTAGGGTGATGGTTCACGTAGTGGG
CCATCGCCCTGATAGACGGTTTTTCGCCCTTTGACGTTGGAGTCCACGTTCTTTAATAGTGGACTCTTGTTCCAAA
CTGGAACAACACTCAACCCTATCTCGGTCTATTCTTTTGATTTATAAGGGATTTTGCCGATTTCGGCCTATTGGTT
AAAAAATGAGCTGATTTAACAAAAATTTAACGCGAATTTTAACAAAATATTAACGTTTACAATTTCAGGTGGCAC
TTTTCGGGGAAATGTGCGCGGAACCCCTATTTGTTTATTTTTCTAAATACATTCAAATATGTATCCGCTCATGAAT
TAATTCTTAGAAAAACTCATCGAGCATCAAATGAAACTGCAATTTATTCATATCAGGATTATCAATACCATATTTT
TGAAAAAGCCGTTTCTGTAATGAAGGAGAAAACTCACCGAGGCAGTTCCATAGGATGGCAAGATCCTGGTATCG
GTCTGCGATTCCGACTCGTCCAACATCAATACAACCTATTAATTTCCCCTCGTCAAAAATAAGGTTATCAAGTGAG
AAATCACCATGAGTGACGACTGAATCCGGTGAGAATGGCAAAAGTTTATGCATTTCTTTCCAGACTTGTTCAACA
GGCCAGCCATTACGCTCGTCATCAAAATCACTCGCATCAACCAAACCGTTATTCATTCGTGATTGCGCCTGAGCG
AGACGAAATACGCGATCGCTGTTAAAAGGACAATTACAAACAGGAATCGAATGCAACCGGCGCAGGAACACTG
CCAGCGCATCAACAATATTTTCACCTGAATCAGGATATTCTTCTAATACCTGGAATGCTGTTTTCCCGGGGATCGC
AGTGGTGAGTAACCATGCATCATCAGGAGTACGGATAAAATGCTTGATGGTCGGAAGAGGCATAAATTCCGTC
AGCCAGTTTAGTCTGACCATCTCATCTGTAACATCATTGGCAACGCTACCTTTGCCATGTTTCAGAAACAACTCTG
GCGCATCGGGCTTCCCATACAATCGAT
AGATTGTCGCACCTGATTGCCCGACATTATCGCGAGCCCATTTATACCCATATAAATCAGCATCCATGTTGGAATT
TAATCGCGGCCTAGAGCAAGACGTTTCCCGTTGAATATGGCTCATAACACCCCTTGTATTACTGTTTATGTAAGC
AGACAGTTTTATTGTTCATGACCAAAATCCCTTAACGTGAGTTTTCGTTCCACTGAGCGTCAGACCCCGTAGAAA
AGATCAAAGGATCTTCTTGAGATCCTTTTTTTCTGCGCGTAATCTGCTGCTTGCAAACAAAAAAACCACCGCTACC
AGCGGTGGTTTGTTTGCCGGATCAAGAGCTACCAACTCTTTTTCCGAAGGTAACTGGCTTCAGCAGAGCGCAGA
TACCAAATACTGTCCTTCTAGTGTAGCCGTAGTTAGGCCACCACTTCAAGAACTCTGTAGCACCGCCTACATACCT
CGCTCTGCTAATCCTGTTACCAGTGGCTGCTGCCAGTGGCGATAAGTCGTGTCTTACCGGGTTGGACTCAAGAC
GATAGTTACCGGATAAGGCGCAGCGGTCGGGCTGAACGGGGGGTTCGTGCACACAGCCCAGCTTGGAGCGAA
CGACCTACACCGAACTGAGATACCTACAGCGTGAGCTATGAGAAAGCGCCACGCTTCCCGAAGGGAGAAAGGC
GGACAGGTATCCGGTAAGCGGCAGGGTCGGAACAGGAGAGCGCACGAGGGAGCTTCCAGGGGGAAACGCCT
GGTATCTTTATAGTCCTGTCGGGTTTCGCCACCTCTGACTTGAGCGTCGATTTTTGTGATGCTCGTCAGGGGGGC
GGAGCCTATGGAAAAACGCCAGCAACGCGGCCTTTTTACGGTTCCTGGCCTTTTGCTGGCCTTTTGCTCACATGT
TCTTTCCTGCGTTATCCCCTGATTCTGTGGATAACCGTATTACCGCCTTTGAGTGAGCTGATACCGCTCGCCGCAG

CCGAACGACCGAGCGCAGCGAGTCAGTGAGCGAGGAAGCGGAAGAGCGCCTGATGCGGTATTTTCTCCTTACG
CATCTGTGCGGTATTTCACACCGCATATATGGTGCACTCTCAGTACAATCTGCTCTGATGCCGCATAGTTAAGCCA
GTATACACTCCGCTATCGCTACGTGACTGGGTCATGGCTGCGCCCCGACACCCGCCAACACCCGCTGACGCGCCC
TGACGGGCTTGTCTGCTCCCGGCATCCGCTTACAGACAAGCTGTGACCGTCTCCGGGAGCTGCATGTGTCAGAG
GTTTTCACCGTCATCACCGAAACGCGCGAGGCAGCTGCGGTAAAGCTCATCAGCGTGGTCGTGAAGCGATTCAC
AGATGTCTGCCTGTTCATCCGCGTCCAGCTCGTTGAGTTTCTCCAGAAGCGTTAATGTCTGGCTTCTGATAAAGC
GGGCCATGTTAAGGGCGGTTTTTTCCTGTTTGGTCACTGATGCCTCCGTGTAAGGGGGATTTCTGTTCATGGGG
GTAATGATACCGATGAAACGAGAGAGGATGCTCACGATACGGGTTACTGATGATGAACATGCCCGGTTACTGG
AACGTTGTGAGGGTAAACAACTGGCGGTATGGATGCGGCGGGACCAGAGAAAAATCACTCAGGGTCAATGCCA
GCGCTTCGTTAATACAGATGTAGGTGTTCCACAGGGTAGCCAGCAGCATCCTGCGATGCAGATCCGGAACATAA
TGGTGCAGGGCGCTGACTTCCGCGTTTCCAGACTTTACGAAACACGGAAACCGAAGACCATTCATGTTGTTGCTC
AGGTCGCAGACGTTTTGCAGCAGCAGTCGCTTCACGTTCGCTCGCGTATCGGTGATTCATTCTGCTAACCAGTAA
GGCAACCCCGCCAGCCTAGCCGGGTCCTCAACGACAGGAGCACGATCATGCGCACCCGTGGGGCCGCCATGCC
GGCGATAATGGCCTGCTTCTCGCCGAAACGTTTGGTGGCGGGACCAGTGACGAAGGCTTGAGCGAGGGCGTGC
AAGATTCCGAATACCGCAAGCGACAGGCCGATCATCGTCGCGCTCCAGCGAAAGCGGTCCTCGCCGAAAATGAC
CCAGAGCGCTGCCGGCACCTGTCCTACGAGTTGCATGATAAAGAAGACAGTCATAAGTGCGGCGACGATAGTC
ATGCCCCGCGCCCACCGGAAGGAGCTGACTGGGTTGAAGGCTCTCAAGGGCATCGGTCGAGATCCCGGTGCCT
AATGAGTGAGCTAACTTACATTAATTGCGTTGCGCTCACTGCCCGCTTTCCAGTCGGGAAACCTGTCGTGCCAGC
TGCATTAATGAATCGGCCAACGCGCGGGGAGAGGCGGTTTGCGTATTGGGCGCCAGGGTGGTTTTTCTTTTCAC
CAGTGAGACGGGCAACAGCTGATTGCCCTTCACCGCCTGGCCCTGAGAGAGTTGCAGCAAGCGGTCCACGCTG
GTTTGCCCCAGCAGGCGAAAATCCTGTTTGATGGTGGTTAACGGCGGGATATAACATGAGCTGTCTTCGGTATC
GTCGTATCCCACTACCGAGATATCCGCACCAACGCGCAGCCCGGACTCGGTAATGGCGCGCATTGCGCCCAGCG
CCATCTGATCGTTGGCAACCAGCATCGCAGTGGGAACGATGCCCTCATTCAGCATTTGCATGGTTTGTTGAAAAC
CGGACATGGCACTCCAGTCGCCTTCCCGTTCCGCTATCGGCTGAATTTGATTGCGAGTGAGATATTTATGCCAGC
CAGCCAGACGCAGACGCGCCGAGACAGAACTTAATGGGCCCGCTAACAGCGCGATTTGCTGGTGACCCAATGC
GACCAGATGCTCCACGCCCAGTCGCGTACCGTCTTCATGGGAGAAAATAATACTGTTGATGGGTGTCTGGTCAG
AGACATCAAGAAATAACGCCGGAACATTAGTGCAGGCAGCTTCCACAGCAATGGCATCCTGGTCATCCAGCGGA
TAGTTAATGATCAGCCCACTGACGCGTTGCGCGAGAAGATTGTGCACCGCCGCTTTACAGGCTTCGACGCCGCTT
CGTTCTACCATCGACACCACCACGCTGGCACCCAGTTGATCGGCGCGAGATTTAATCGCCGCGACAATTTGCGAC
GGCGCGTGCAGGGCCAGACTGGAGGTGGCAACGCCAATCAGCAACGACTGTTTGCCCGCCAGTTGTTGTGCCA
CGCGGTTGGGAATGTAATTCAGCTCCGCCATCGCCGCTTCCACTTTTTCCCGCGTTTTCGCAGAAACGTGGCTGG
CCTGGTTCACCACGCGGGAAACGGTCTGATAAGAGACACCGGCATACTCTGCGACATCGTATAACGTTACTGGT
TTCACATTCACCACCCTGAATTGACTCTCTTCCGGGCGCTATCATGCCATACCGCGAAAGGTTTTGCGCCATTCGA
TGGTGTCCGGGATCTCGACGCTCTCCCTTATGCGACTCCTGCATTAGGAAGCAGCCCAGTAGTAGGTTGAGGCC
GTTGAGCACCGCCGCCGCAAGGAATGGTGCATGCAAGGAGATGGCGCCCAACAGTCCCCCGGCCACGGGGCCT
GCCACCATACCCACGCCGAAACAAGCGCTCATGAGCCCGAAGTGGCGAGCCCGATCTTCCCCATCGGTGATGTC
GGCGATATAG

>pPG20

GGCCGCGGTACCAGATCTTTAAATCTAGATATCCATGGCTCTCACACCCCCTCTTCCATTCTGGCACTCGATGCCA
TATATTTGCGATCTCGATCACAACTGTCGAGACCATACGCGAGAATTCGACCGGAACGGATTGCTGACATGACC
GGACCGAGACTGCCCGACGGTTTTGCCGTGCAGGTGGATCGCCGAGTCAAGGTGCTCGGAGAGGGCGCGGCG
TTGCTGGGCGGCTCACCGACCCGCCTGCTGCGGTTGGCGCCGACGGCCCAGAACATGCTCAGCGGGGGCCGCC
TCGAGGTTCACGACGCGGTGTCCGCGCAACTCGCGCGAACCCTCCTGGACGCGACCGTCGCGCATCCGCGGCCT
GCCAGCGGGCCGTCCCATCTGGACGTCACAGTGGTTGTCCCCGTACGGGACAACGCATCCGGCCTGCACCGCCT
GATGGCGGCGCTGCGCGGGCTGCGCGTGATCGTGGTCGACGACGGCTCGGCGATCCCGGTGCAACCGTCCGAC
TTCTCCGGTATGCACTGCGACGTGCAGGTGCTGCGGCACACCCGCAGCAACGGCCCCGCGGCCGCGCGTAACAC
CGGCCTTGCGTCGTGCGAGACGGATTTCGTGGCGTTCCTCGATTCCGACGTGGTGCCCAAGCGTGGTTGGCTCG
AGGCGCTGCTCGGGCATTTCTGCGATCCGGCCGTGGCACTGGTGGCGCCCCGCATCGTGGGTCTGCACAACGCC
GACAACATCGTGGCCCGGTACGAGTCCGTCCGATCCTCGCTGGACCTCGGGGTGCGCGAGGCGCCCGTGGTGC
CGCACGGCACGGTGTCGTATGTGCCGAGCGCGGCGATCATCTGCAGGCGGTCGGCGCTCGTGGAAGTGGGCG
GGTTCGACGAGACCATGCACTCGGGGGAGGATGTCGACCTGTGCTGGCGCCTGGTCGAGTCGGGCGCGCGTCT
GCGTTACGAACCGATCGCCCTGGTGGCGCACGATCACCGCACCAACCTGCGAGCGTGGTTCCACCGCAAGGCAT
TCTATGGAACGTCGGCCGCTCCGCTGACGGTGCGCCATCCCGGTAAGACGTCGCCGCTGGTGATCTCGGGGTG
GACGCTGATGGTGTGGCTGATGCTCGGGGTGGGCTCGTTCTTCGGCTACCTCGCCTCGCTCGCGGCGGCGGTGT
TCGCGGGTACCCGCATCGCGCGGGCGCTCAGCGTCGTCGAGACCGAACCCAAGGAGGTCGCGGTGGTCGCCGC
CCACGGCCTGTGGTCGTCGGCGTTGCAGTTGTGTTCGGCGATCTGTCGCCACTACTGGCCCATCGCGATGATCG
CGGCGGTGCTGTTCCGCCGGGCGCGGCACGCGGTGCTGGTGGCCGCGGTGGTGGACGGTGTGGTCGACTGGG
TGACGCGACGCGGCAACGCCGACGACGACACCAAACCGGTCGGACTGCTCACCCACATCGTGCTCAAGCGACT
GGACGACATCGCCTACGGCACCGGTCTGTGGACCGGCGTGGTGCGCGAGCGTCACCTCGGCGCGCTCAAGCCC
CAGGTGCGGAGTTAGAAGCTTATCGATGTCGACGTAGTTAACTAGCGTACGATCGACTGCCAGGCATCAAATAA
AACGAAAGGCTCAGTCGAAAGACTGGGCCTTTCGTTTTATCTGTTGTTTGTCCGGCCATCATGGCCGCGGTGATC
AGCTAGAGCCGTGAACGACAGGGCGAACGCCAGCCCGCCGACGGCGAGGGTTCCGACCGCTGCAACTCCCGGT
GCAACCTTGTCCCGGTCTATTCTCTTCACTGCACCAGCTCCAATCTGGTGTGAATGCCCCTCGTCTGTTCGCGCAG
GCGGGGGGCTCTATTCGTTTGTCAGCATCGAAAGTAGCCAGATCAGGGATGCGTTGCAACCGCGTATGCCCAG
GTCAGAAGAGTCGCACAAGAGTTGCAGACCCCTGGAAAGAAAAATGGCCAGAGGGCGAAAACACCCTCTGACC
AGCGGAGCGGGCGACGGGAATCGAACCCGCGTAGCTAGTTTGGAAGAATGGGTGTCTGCCGACCACATATATG
GGCCGGTCAAGATAGGTTTTTACCCCCTCTCGGCTGCATCCTCTAAGTGGAAAGAAATTGCAGGTCGTAGAAGC
GCGTTGAAGCCTGAGAGTTGCACAGGAGTTGCAACCCGGTAGCCTTGTTCACGACGAGAGGAGACCTAGTTGG
CACGTCGCGGATGGGGATCGCTGAAGACTCAGCGCAGCGGGAGGATCCAAGCCTCATACGTCAACCCGCAGGA
CGGTGTGAGGTACTACGCGCTGCAGACCTACGACAACAAGATGGACGCCGAAGCCTGGCTCGCGGGCGAGAA
GCGGCTCATCGAGATGGAGACCTGGACCCCTCCACAGGACCGGGCGAAGAAGGCAGCCGCCAGCGCCATCACG
CTGGAGGAGTACACCCGGAAGTGGCTCGTGGAGCGCGACCTCGCAGACGGCACCAGGGATCTGTACAGCGGG
CACGCGGAGCGCCGCATCTACCCGGTGCTAGGTGAAGTGGCGGTCACAGAGATGACGCCAGCTCTGGTGCGTG
CGTGGTGGGCCGGGATGGGTAGGAAGCACCCGACTGCCCGCCGGCATGCCTACAACGTCCTCCGGGCGGTGAT
GAACACAGCGGTCGAGGACAAGCTGATCGCAGAGAACCCGTGCCGGATCGAGCAGAAGGCAGCCGATGAGCG
CGACGTAGAGGCGCTGACGCCTGAGGAGCTGGACATCGTCGCCGCTGAGATCTTCGAGCACTACCGGATCGCG
GCATACATCCTGGCGTGGACGAGCCTCCGGTTCGGAGAGCTGATCGAGCTTCGCCGCAAGGACATCGTGGACG
ACGGCATGACGATGAAGCTCCGGGTGCGCCGTGGCGCTTCCCGCGTGGGGAACAAGATCGTCGTTGGCAACGC
CAAGACCGTCCGGTCGAAGCGTCCTGTGACGGTTCCGCCTCACGTCGCGGAGATGATCCGAGCGCACATGAAG
GACCGTACGAAGATGAACAAGGGCCCCGAGGCATTCCTGGTGACCACGACGCAGGGCAACCGGCTGTCGAAGT
CCGCGTTCACCAAGTCGCTGAAGCGTGGCTACGCCAAGATCGGTCGGCCGGAACTCCGCATCCACGACCTCCGC
GCTGTCGGCGCTACGTTCGCCGCTCAGGCAGGTGCGACGACCAAGGAGCTGATGGCCCGTCTCGGTCACACGA
CTCCTAGGATGGCGATGAAGTACCAGATGGCGTCTGAGGCCCGCGACGAGGCTATCGCTGAGGCGATGTCCAA
GCTGGCCAAGACCTCCTGAAACGCAAAAAGCCCCCCTCCCAAGGACACTGAGTCCTAAAGAGGGGGGTTTCTTG
TCAGTACGCGAAGAACCACGCCTGGCCGCGAGCGCCAGCACCGCCGCTCTGTGCGGAGACCTGGGCACCAGCC
CCGCCGCCGCCAGGAGCATTGCCGTTCCCGCCAGCTAGCAACAAAGCGACGTTGTGTCTCAAAATCTCTGATGTT

ACATTGCACAAGATAAAAATATATCATCATGAACAATAAAACTGTCTGCTTACATAAACAGTAATACAAGGGGTG
TTATGAGCCATATTCAACGGGAAACGTCTTGCTCGAGGCCGCGATTAAATTCCAACATGGATGCTGATTTATATG
GGTATAAATGGGCTCGCGATAATGTCGGGCAATCAGGTGCGACAATCTATCGCTTGTATGGGAAGCCCCATGCG
CCAGAGTTGTTTCTGAAACATGGCAAAGGTAGCGTTGCCAATGATGTTACAGATGAGATGGTCAGACTAAACTG
GCTGACGGAATTTATGCCTCTTCCGACCATCAAGCATTTTATCCGTACTCCTGATGATGCATGGTTACTCACCACT
GCGATCCCCGGGAAAACAGCATTCCAGGTATTAGAAGAATATCCTGATTCAGGTGAAAATATTGTTGATGCGCT
GGCAGTGTTCCTGCGCCGGTTGCATTCGATTCCTGTTTGTAATTGTCCTTTTAACAGCGATCGCGTATTTCGTCTC
GCTCAGGCGCAATCACGAATGAATAACGGTTTGGTTGATGCGAGTGATTTTGATGACGAGCGTAATGGCTGGCC
TGTTGAACAAGTCTGGAAAGAAATGCATAATCTTTTGCCATTCTCACCGGATTCAGTCGTCACTCATGGTGATTTC
TCACTTGATAACCTTATTTTTGACGAGGGGAAATTAATAGGTTGTATTGATGTTGGACGAGTCGGAATCGCAGAC
CGATACCAGGATCTTGCCATCCTATGGAACTGCCTCGGTGAGTTTTCTCCTTCATTACAGAAACGGCTTTTTCAAA
AATATGGTATTGATAATCCTGATATGAATAAATTGCAGTTTCATTTGATGCTCGATGAGTTTTTCTAATCAGAATT
GGTTAATTGGTTGTAACACTGGCAGAGCATTACGCTGACTTGACGGGACGGCGGCTTTGTTGAATAAATCGAAC
TTTTGCTGAGTTGAAGGATCAGATCACGCATCTTCCCGACAACGCAGACCGTTCCGTGGCAAAGCAAAAGTTCA
AAATCACCAACTGGTCCACCTACAACAAAGCTCTCATCAACCGTGGCTCCCTCACTTTCTGGCTGGATGATGGGG
CGATTCAGGCCTGGTATGAGTCAGCAACACCTTCTTCACGAGGCAGACCTCACTAGTTCCACTGAGCGTCAGACC
CCGTAGAAAAGATCAAAGGATCTTCTTGAGATCCTTTTTTTCTGCGCGTAATCTGCTGCTTGCAAACAAAAAAAC
CACCGCTACCAGCGGTGGTTTGTTTGCCGGATCAAGAGCTACCAACTCTTTTTCCGAAGGTAACTGGCTTCAGCA
GAGCGCAGATACCAAATACTGTCCTTCTAGTGTAGCCGTAGTTAGGCCACCACTTCAAGAACTCTGTAGCACCGC
CTACATACCTCGCTCTGCTAATCCTGTTACCAGTGGCTGCTGCCAGTGGCGATAAGTCGTGTCTTACCGGGTTGG
ACTCAAGACGATAGTTACCGGATAAGGCGCAGCGGTCGGGCTGAACGGGGGGTTCGTGCACACAGCCCAGCTT
GGAGCGAACGACCTACACCGAACTGAGATACCTACAGCGTGAGCATTGAGAAAGCGCCACGCTTCCCGAAGGG
AGAAAGGCGGACAGGTATCCGGTAAGCGGCAGGGTCGGAACAGGAGAGCGCACGAGGGAGCTTCCAGGGGG
AAACGCCTGGTATCTTTATAGTCCTGTCGGGTTTCGCCACCTCTGACTTGAGCGTCGATTTTTGTGATGCTCGTCA
GGGGGGCGGAGCCTATGGAAAAACGCCAGCAACGCGGCCTTTTTACGGTTCCTGGCCTTTTGCTGGCCTTTTGC
TCACATGTTCTTTCCTGCGTTATCCCCTGATTCTGTGGATAACCGTATTACCGCCTTTGAGTGAGCTGATACCGCT
CGCCGCAGCCGAACGACCGAGCGCAACGCGTGC

References

Ayikpoe RS, Latham JA (2019) MftD catalyzes the formation of a biologically active redox center in the biosynthesis of the ribosomally synthesized and post-translationally modified redox cofactor, mycofactocin. J Am Chem Soc 141: 13582-13591

Berney M, Weimar MR, Heikal A, Cook GM (2012) Regulation of proline metabolism in mycobacteria and its role in carbon metabolism under hypoxia. Mol Microbiol 84: 664-81

Chambers MC, Maclean B, Burke R, Amodei D, Ruderman DL, Neumann S, Gatto L, Fischer B, Pratt B, Egertson J, Hoff K, Kessner D, Tasman N, Shulman N, Frewen B, Baker TA, Brusniak MY, Paulse C, Creasy D, Flashner L et al. (2012) A cross-platform toolkit for mass spectrometry and proteomics. Nat Biotechnol 30: 918-20

Edgar RC (2004) MUSCLE: multiple sequence alignment with high accuracy and high throughput. Nucleic Acids Res 32: 1792-7

Krishnamoorthy G, Kaiser P, Lozza L, Hahnke K, Mollenkopf HJ, Kaufmann SHE (2019) Mycofactocin is associated with ethanol metabolism in mycobacteria. mBio 10

Krogh A, Larsson B, von Heijne G, Sonnhammer EL (2001) Predicting transmembrane protein topology with a hidden Markov model: application to complete genomes. J Mol Biol 305: 567-80

Lombard V, Golaconda Ramulu H, Drula E, Coutinho PM, Henrissat B (2014) The carbohydrate-active enzymes database (CAZy) in 2013. Nucleic Acids Res 42: D490-5

Shannon P, Markiel A, Ozier O, Baliga NS, Wang JT, Ramage D, Amin N, Schwikowski B, Ideker T (2003) Cytoscape: a software environment for integrated models of biomolecular interaction networks. Genome Res 13: 2498-504

van der Werf MJ, van der Ven C, Barbirato F, Eppink MH, de Bont JA, van Berkel WJ (1999) Stereoselective carveol dehydrogenase from *Rhodococcus erythropolis* DCL14. A novel nicotinoprotein belonging to the short chain dehydrogenase/reductase superfamily. J Biol Chem 274: 26296-304

Wang M, Carver JJ, Phelan VV, Sanchez LM, Garg N, Peng Y, Nguyen DD, Watrous J, Kapono CA, Luzzatto-Knaan T, Porto C, Bouslimani A, Melnik AV, Meehan MJ, Liu WT, Crusemann M, Boudreau PD, Esquenazi E, Sandoval-Calderon M, Kersten RD et al. (2016) Sharing and community curation of mass spectrometry data with Global Natural Products Social Molecular Networking. Nat Biotechnol 34: 828-837

Warner DF, Ndwandwe DE, Abrahams GL, Kana BD, Machowski EE, Venclovas C, Mizrahi V (2010) Essential roles for *imuA*'- and *imuB*-encoded accessory factors in DnaE2-dependent mutagenesis in *Mycobacterium tuberculosis*. Proc Natl Acad Sci U S A 107: 13093-8
